# Dual-ROS-scavenging and dual-lingering nanozyme-based eye drops alleviate dry eye disease

**DOI:** 10.1186/s12951-024-02499-0

**Published:** 2024-05-08

**Authors:** Wei Zhang, Mengyang Zhao, Dandan Chu, Huiying Chen, Bingbing Cui, Qingyun Ning, Xing Wang, Zhanrong Li, Shaokui Cao, Jingguo Li

**Affiliations:** 1 Henan Eye Hospital, Henan Provincial People’s Hospital, People’s Hospital of Zhengzhou University, Zhengzhou, 450003 China; 2 School of Material Science and Engineering, Zhengzhou University, Zhengzhou, 450001 China

**Keywords:** Dry eye disease, Nanozyme eye drops, Reactive oxygen species, Boronic ester bonding, Nanomedicine

## Abstract

Efficiently removing excess reactive oxygen species (ROS) generated by various factors on the ocular surface is a promising strategy for preventing the development of dry eye disease (DED). The currently available eye drops for DED treatment are palliative, short-lived and frequently administered due to the short precorneal residence time. Here, we developed nanozyme-based eye drops for DED by exploiting borate-mediated dynamic covalent complexation between n-FeZIF-8 nanozymes (n-Z(Fe)) and poly(vinyl alcohol) (PVA) to overcome these problems. The resultant formulation (PBnZ), which has dual-ROS scavenging abilities and prolonged corneal retention can effectively reduce oxidative stress, thereby providing an excellent preventive effect to alleviate DED. In vitro and in vivo experiments revealed that PBnZ could eliminate excess ROS through both its multienzyme-like activity and the ROS-scavenging activity of borate bonds. The positively charged nanozyme-based eye drops displayed a longer precorneal residence time due to physical adhesion and the dynamic borate bonds between phenyboronic acid and PVA or o-diol with mucin. The in vivo results showed that eye drops could effectively alleviate DED. These dual-function PBnZ nanozyme-based eye drops can provide insights into the development of novel treatment strategies for DED and other ROS-mediated inflammatory diseases and a rationale for the application of nanomaterials in clinical settings.

**Graphical Abstract:**

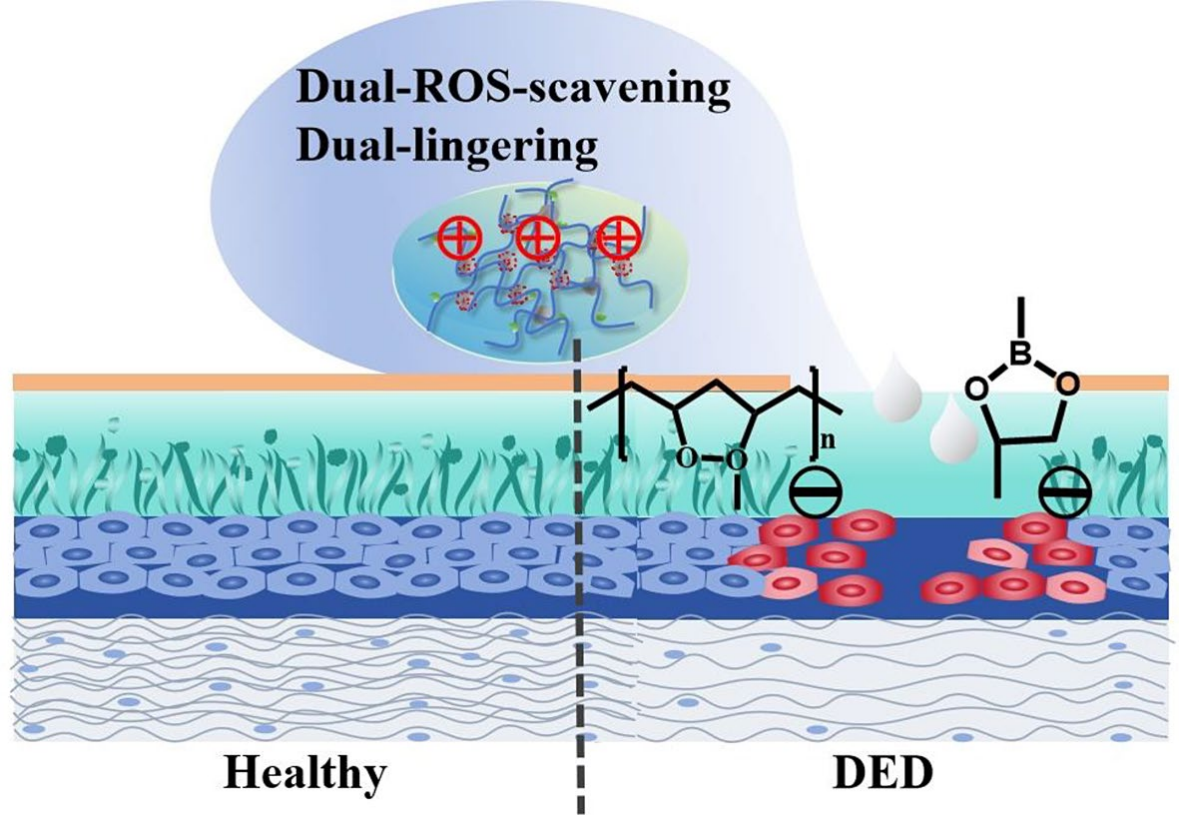

**Supplementary Information:**

The online version contains supplementary material available at 10.1186/s12951-024-02499-0.

## Highlights

Modification of dynamic covalent bonds based on borate bonds can improve the solubility and dispersion of Fe-based nanozymes in solution.

The nanozyme-based eye drops enable reactive groups to bind to the chemical groups present in the mucin layer to confer carrier adhesion, thereby increasing the retention time of the carrier on the ocular surface.

Nanozyme-based eye drops have synergistic effects on Fe-based nanozymes, and borate bonds effectively neutralize excess ROS in the microenvironment of the ocular surface, thereby reducing oxidative stress and direct oxidative damage.

Nanozyme-based eye drops could effectively alleviate DED and may be a promising nanotherapeutic strategy for DED treatment.

**Supplementary Information:**

The online version contains supplementary material available at 10.1186/s12951-024-02499-0.

## Introduction

Dry eye disease (DED) is a multifactorial ocular disease affecting millions of people worldwide. DED is characterized by changes in the composition and stability of the tear film, epithelial cell dysfunction, and goblet cell loss in the conjunctiva, which can lead to increased tear osmolarity and inflammation.[1] Oxidative stress is closely related to the occurrence of DED. Exposure to oxidative stress on the ocular surface leads to excessive production of associated reactive oxygen species (ROS), such as hydrogen peroxide (H_2_O_2_) and superoxide anion (O_2_^•−^), resulting in disturbances in cellular metabolism and the destruction of cellular components.[2] In a normal biological environment, ROS play an important role in redox regulation and are balanced by the biological antioxidant system; however, chronic exposure to oxidative stress leads to excess production of ROS, and the antioxidant defense system cannot sufficiently counteract the excessively generated ROS.[3] ROS can accumulate in the tear membrane and cornea of DED patients, leading to impaired lacrimal gland function. The cornea is located in the anterior region of the eye and is directly exposed to ultraviolet light, radiation air pollution, and blue light from mobile devices, resulting in the overproduction of ROS and oxidative stress injury to the ocular surface.[4] In addition, oxidative stress can lead to the overexpression of the proinflammatory cytokines interleukin-1β (IL-1β) and interleukin-6 (IL-6) in immune cells, which causes inflammation on the ocular surface and tear film hyperpermeability and may ultimately lead to DED.[5] Therefore, clearing overproduced pathological ROS may constitute a novel strategy for alleviating DED.

Recently, treatment methods for DED have focused on relieving symptoms, reducing ocular surface inflammation and improving the stability of the tear film, however, these drugs can only temporarily relieve symptoms by wetting and lubricating the ocular surface and cannot fundamentally relieve DED.[6] The development of nanomaterials with enzymatic catalytic properties, including metal-based nanoparticles, nanocarbon/selenium, and polyphenol nanoformulations, has attracted great attention.[7] Artificial bionic nanomaterials termed nanozymes can be designed to mimic natural antioxidant enzymes for scavenging multiple types of reactive species, including superoxide dismutase (SOD), catalase (CAT), and peroxidase (POD).[8] Compared to small molecule antioxidant drugs and natural enzymes, nanozymes not only exhibit the ability to eliminate ROS and inhibit inflammation but also possess unique advantages, such as low cost, high stability, resistance to harsh environments, suitable biocompatibility, and programmability.[9] Iron (Fe) is a transition metal-based element that serves as the active center of many natural antioxidant enzymes. The electron transfer reaction of the Fe(III)/Fe(II) complex functionally helps to eliminate oxidative stress and restore the balance between ROS production and antioxidant enzymes.[10] However, the catalytic activity of Fe-based nanozymes toward ROS depends on the morphology, particle size, and ion ratio of surface Fe^3+^ to Fe^2+^. Although Fe-based nanozymes can be used to treat ocular surface diseases, further clinical application of these materials is limited by their potential toxicity and relatively low dispersion solubility, relative stability and catalytic activity.[11] Therefore, studies with the aim of identifying effective and direct modulation strategies to improve the efficacy and safety of Fe-based nanozymes are urgently needed.

Dynamic covalent bonds are a class of chemical bonds that can be reversibly broken and reformed under external conditions (such as light, pH, and catalysis);[12] they have attracted wide interest due to their ability to release drugs, act as cellular adaptive scaffolds, and respond to stimuli in the physiological microenvironment.[13] In treating ocular surface diseases, the introduction of a mildly reactive functional group enables drug agents to bind to the chemical groups present in the mucin layer through dynamic covalent bonds, thereby increasing the carrier and the retention time of the carrier on the ocular surface.[14] For example, boronic acids are used in a myriad of synthesis processes. Phenylboronic acid (PBA), a mild Lewis acid, is a commonly used boronic acid that bonds well with o-diol-carrying substances via reversible covalent bonds.[15] Dynamic covalent complexation between cis-diol and boronic acid moieties has been utilized in numerous biomedical applications; these moieties can bind to cis-hydroxyl groups on receptors such as glycoproteins on cell membranes.[16] Because of its high affinity for ethylene glycol, PBA-modified micelles can adhere to the ocular surface by forming a dynamic chemical bond with the o-diol in mucin. Furthermore, PBA can ionize with free hydrogen and oxygen and become an anion with a negative charge, which results in a strong retention ability on the surface of anions such as those in mucin under such conditions.[17] Importantly, the formation and dissociation of boronic ester derivatives exhibit wide application potential by concurrently exhibiting enhanced ROS sensitivity. The borate bond is prone to break under excess ROS conditions and is oxidized to phenol and boric acid, while phenol can be further oxidized.[18] Therefore, since a variety of reaction methods for PBA have been developed, more choices are available for the design of PBA-based materials, which can increase their performance.

**Scheme 1:**
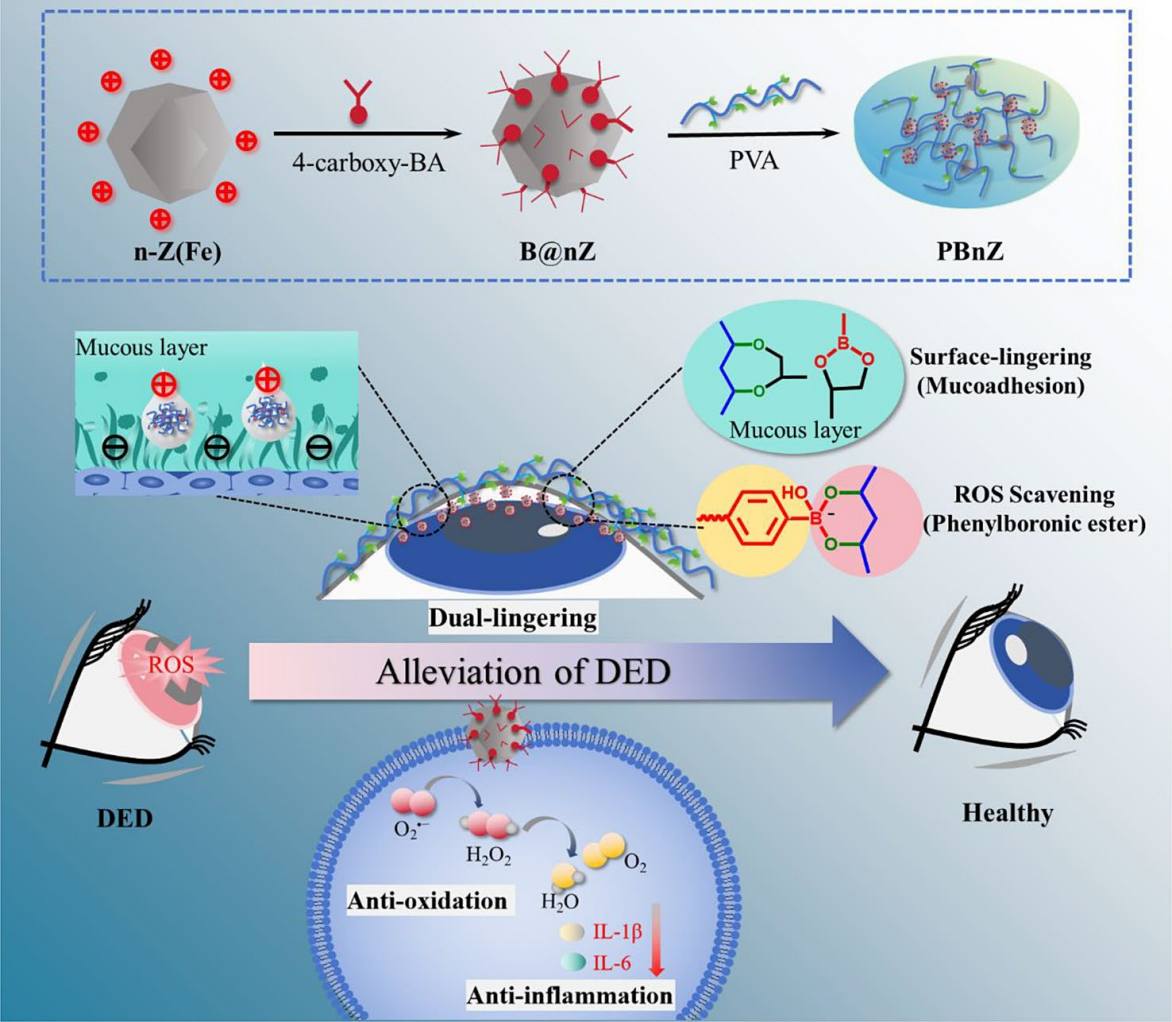
Schematic diagram of the synthesis of PBnZ nanozyme eye drops and PBnZ with dual ROS-scavenging and dual-lingering abilities that can increase ocular retention and continuously scavenge ROS to alleviate DED

In this study, we developed nanozyme-based eye drops for DED alleviation by grafting 4-carboxy-phenylboronic acid (PBA) to the amine group of Fe-based nanozymes through an acylation reaction and cross-linking with poly(vinyl alcohol) (PVA). The resultant formulation (PBnZ) was designed to scavenge ROS, extend ocular retention, and provide an excellent preventive effect against DED (Scheme 1). The dynamic borate bonds were modified by an acylation reaction to improve the solubility and dispersion of Fe-based nanozymes in solution. The synergistic action of Fe-based nanozymes and borate bonds effectively neutralized excess ROS in the microenvironment of the ocular surface, thereby reducing oxidative stress and direct oxidative damage. The combination of PBA or PVA with the mucin layer increased the ocular surface retention time. Therefore, this study aimed to investigate the role of PBnZ in DED and its underlying mechanism.

## Results and discussion

### Preparation and characterization of PBnZ

**Fig. 1:**
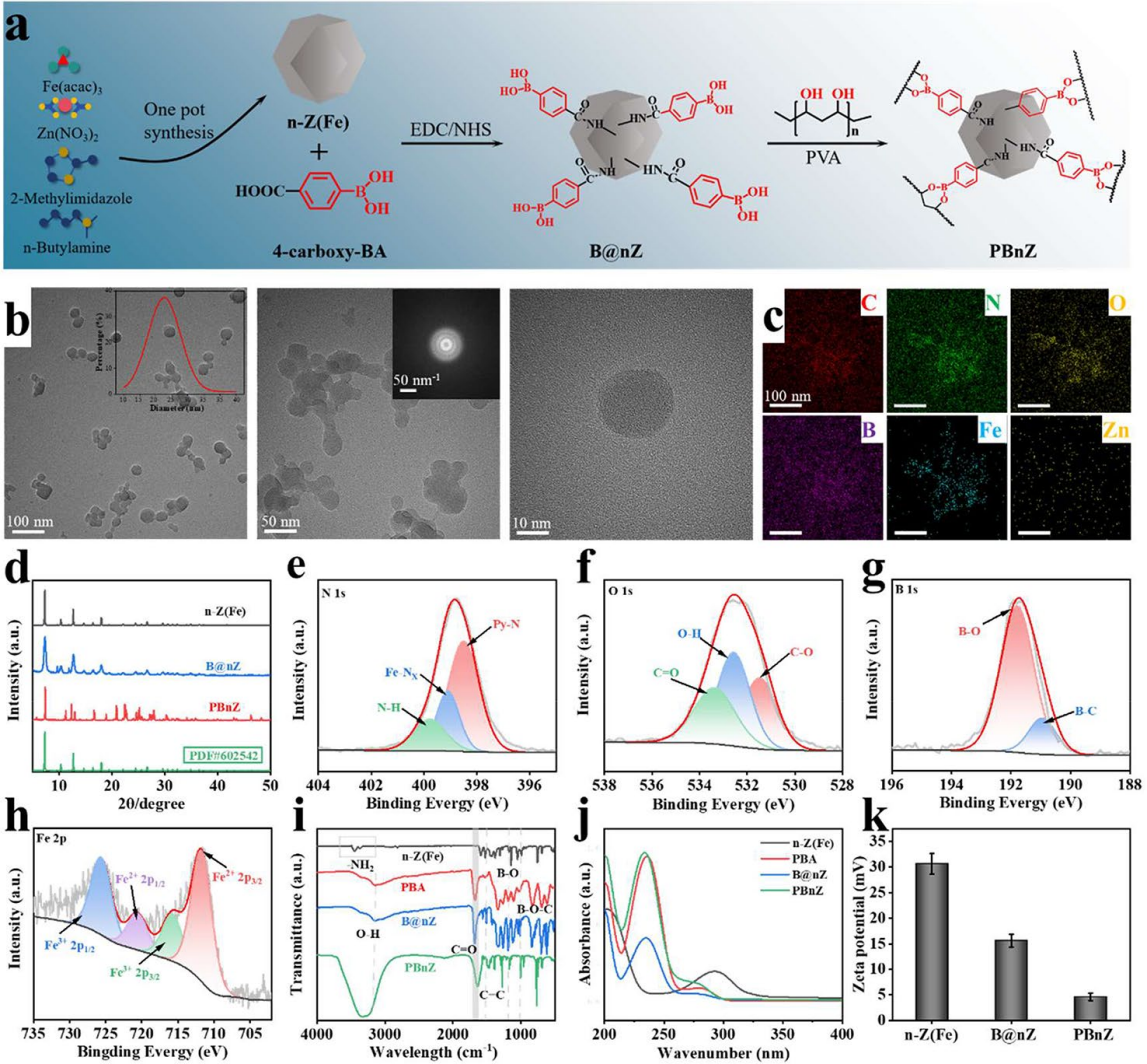
Design and characterization of PBnZ. (**a**) Schematic illustration of the mechanism of PBnZ synthesis. (**b**) Representative TEM image and SAED pattern of PBnZ. Inset: Particle size distribution of PBnZ. (**c**) Corresponding EDS mappings of PBnZ showing that C, N, O, B, Fe and Zn were evenly distributed in the material. (**d**) XRD patterns of n-Z(**Fe**), B@n-Z and PBnZ. (**e**)-(**h**) XPS spectra of PBnZ: (**e**) N *1s*, (**f**) O *1s*, (**g**) B *1s* and (**h**) Fe *2p*. (**i**) FTIR spectra of n-Z(**Fe**), PBA, B@nZ and PBnZ. (**j**) UV‒vis spectra of n-Z(**Fe**), PBA, B@nZ and PBnZ. (**k**) Zeta potentials of n-Z(**Fe**), B@nZ and PBnZ (*n* = 3 independent experiments)

PBnZ was prepared using a three-step method. First, as depicted in Fig. 1a, n-Z(Fe) nanozymes were synthesized via a one-pot hydrothermal method by reacting Zn(NO_3_)_2_·6H_2_O, Fe(acac)_3_, n-butylamine and 2-methylimidazole in methanol. C, N, O, Fe and Zn were homogeneously distributed according to energy dispersive X-ray spectrometry (EDS) elemental mapping (Fig. S1). Second, the resulting n-Z(Fe) was further dispersed in pure water in the presence of PBA, N-hydroxy succinimide (NHS) and 1-ethyl-3-(3-dimethylaminopropyl) carbodiimide hydrochloride (EDC) to yield the desired B@nZ. Finally, B@nZ was dispersed in artificial tears through an ultrasonic treatment, the pH was adjusted to 8.5, PVA (1% w/v) was added to the dispersion, The 1% w/v PVA was chosen because it was consistent with the concentration of commercially avaiable eye drops. The final product, nanozyme-based eye drops, was obtained (named PBnZ). The surface morphology of PBnZ was examined via transmission electron microscopy (TEM) combined with EDS. The average size of the PBnZ particles was 25 nm, as shown in their TEM images (Fig. 1b), which included some isolated particles and particles with sharp edges. Notably, small ZIF nanocrystals are very sensitive to the high energy of the electron beam used in TEM.[19] After the introduction of n-butylamine, the ligand can be used for the regulation of complex formation and deprotonation equilibrium during crystal nucleation and growth. Although nucleation and growth are not separate, in the early stage, the particle size distribution of monodisperse nanocrystals and the prepared PBnZ nanomaterials are stable, and this small size makes PBnZ suitable for DED therapy. Selected area electron diffraction (SAED) patterns confirmed that the particles were crystalline PBnZ. Furthermore, corresponding EDS elemental mappings revealed that the surface of PBnZ contained large amounts of C, N and O and a uniform distribution of B, Fe and Zn (Fig. 1c). This result confirmed that the successful formation of PBnZ and PBA on the surface of n-Z(Fe) was not only a result of their physical action.

X-ray diffraction (XRD) and X-ray photoelectron spectroscopy (XPS) were also conducted to analyze the surface chemical properties and the crystal structure of PBnZ. The XRD patterns showed that PBnZ had the same characteristic peaks as n-Z(Fe), B@nZ and the reported FeZIF-8 (PDF#602,542)[20], indicated the successful formation of PBnZ without altering its crystallinity. Furthermore, the diffraction peak widened as the stock concentration decreased, indicated that fewer particles were produced, a finding consistent with the particle size results (Fig. 1d). This finding also showed that PBnZ had a characteristic peak and high crystallinity. Moreover, as evidenced by the XPS analysis, the chemical bond peaks of PBnZ located at 190 eV, 284 eV, 399 eV, 532 eV, 720 eV and 1020 eV resulted from B *1s*, C *1s*, N *1s*, O *1s*, Fe 2*p* and Zn 2*p*, respectively (Fig. S2). The C *1s* diffraction peaks of PBnZ were deconvoluted into C = C bonds, C–N bonds, and C = O bonds at 284.3, 284.9, 285.5, and 288.9 eV, respectively (Fig. S3). The N *1s* spectrum of PBnZ was deconvoluted into three subpeaks at 398.5, 399.1 and 400.24 eV, which were attributed to pyrrolic N, Fe-Nx, and N-H groups, respectively (Fig. 1e). The O *1s* XPS spectrum in Fig. 1f was deconvoluted into three subpeaks at 532.4, 536.5 and 538.8 eV, which were attributed to C-O, O-H and C = O groups, respectively. The B *1s* spectrum of PBnZ was deconvoluted into two individual peaks at 190.08 eV and 191.54 eV (Fig. 1g), which could be assigned to the B-C and B-O peaks, respectively. The Zn *2p* spectrum could be attributed to two main contributions, *2p*_*3/2*_ and 2*p*_*1/2*_, which are located at 1021.8 eV and 1044.8 eV, respectively (Fig. S4). The presence of Fe in PBnZ was also validated by the presence of several Fe peaks, including Fe(II) 2*p*_*3/2*_ (710.2 eV), Fe(III) 2*p*_*3/2*_ (713.8 eV), Fe(II) *2p*_*1/2*_ (723.4 eV), and Fe(III) *2p*_*1/2*_ (726.7 eV) (Fig. 1h). Functional groups containing O and N can interact with the ocular surface mucin layer through reactions such as complexation and electrostatic attraction to enhance the long-term retention of the ocular surface. Taken together, these results indicated that a borate ester was successfully introduced into n-Z(Fe) to enable ROS responsiveness. The various valence changes of Fe also provide favorable conditions for the redox process.

The Fourier-transform infrared (FTIR) spectra of n-Z(Fe), PBA, B@nZ and PBnZ were further characterized (Fig. 1i). After the introduction of PBA, the bands at 1045, 1363, 1725, 1514, and 3125 cm^–1^ were attributed to borate ester B-O-C stretching, B-O units, C = C units, the vibration of the C = O bond and the antisymmetric stretching vibration of O-H, respectively. Similarly, we demonstrated the successful synthesis of B@nZ using a UV‒visible spectrophotometer (UV‒vis). PBA, B@nZ and PBnZ showed UV‒vis absorption peaks at 240 nm, while n-Z(Fe) had no UV‒vis absorption peak, which indicated that phenylboronic acid was successfully modified to n-Z(Fe) (Fig. 1j).

Next, the prepared PBnZ was characterized based on the properties of the drops to determine whether it met the eye drops property criteria for use on the eye surface. The porosity of PBnZ was evaluated by performing nitrogen adsorption–desorption studies. The specific surface area of PBnZ was 1678.6761 m²/g, and the pore size was 17.7497 nm. The surface area of PBnZ was determined to be 8.97 m^2^/g using the Brunauer‒Emmett‒Teller method (Fig. S5). At a certain shear rate, the viscosity of PBnZ was similar to that of PVA and more suitable for ocular surface retention (Fig. S6). These results verified the formation of a large cavity in PBnZ. The zeta potentials of n-Z(Fe), B@nZ and PBnZ were 30 ± 2.6, 15 ± 1.2 and 6 ± 1.2 mV, respectively (Fig. 1k). The zeta potential of PBnZ decreased slightly after PBA modification. The positive charge of PBnZ is more conducive to prolonging the ocular surface retention time and increasing cellular uptake.

Moreover, the hydrophilicity of PBnZ was investigated by conducting water contact angle measurements. As shown in Fig. S7, the nanozymes had enhanced hydrophilicity (49.5 ± 1.5) compared with the water control (69.6 ± 2.5). Subsequently, PBnZ was incubated with artificial tears, saline, or Dulbecco’s modified Eagle medium (DMEM) for 48 h to investigate the dispersion and stability of the nanozymes in different solvents (Fig. S8). No obvious precipitation was detected under the aforementioned conditions, but under 660 nm beam irradiation, clear bright paths were observed, indicating that basic stability could be maintained (Fig. S9). Through the characterization described above, we confirmed the successful preparation of PBnZ, which was further applied in subsequent experiments.

### Multienzyme-like activity and ROS-scavenging activity of PBnZ

**Fig. 2:**
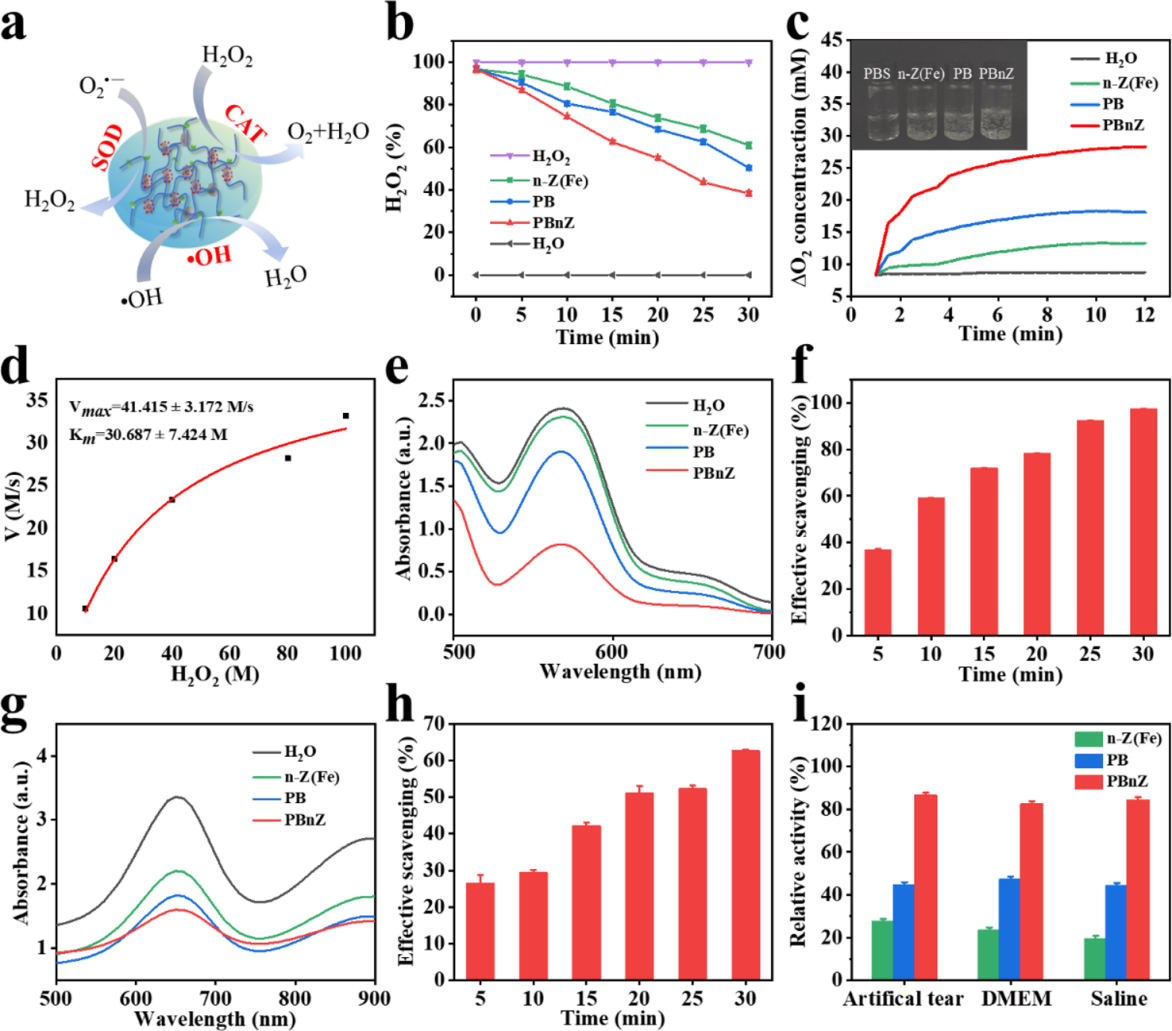
Multienzyme-like antioxidant activity of PBnZ. (**a**) Schematic diagram of the reaction catalyzed by the three-species enzyme. (**b**) H_2_O_2_ decomposition efficiency of H_2_O_2_, n-Z(Fe), PB, PBnZ and H_2_O. (**c**) The CAT-like activity of n-Z(Fe), PB and PBnZ. (**d**) Michaelis‒Menten kinetic analysis of PBnZ with H_2_O_2_ as a substrate. (**e**) Absorption spectra of INT after reaction O_2_^•−^  in the presence of n-Z(Fe), PB and PBnZ. (**f**) SOD-like activities of PBnZ at a concentration of 200 µg/mL (*n* = 3). (**g**) Absorption spectra of TMB after reaction •OH in the presence of n-Z(Fe), PB and PBnZ. (**h**) •OH scavenging activity of PBnZ at a concentration of 200 µg/mL (*n* = 3). (**i**) Relative catalytic stability of n-Z(Fe), PB and PBnZ in artificial tears, saline, and DMEM (containing 10% FBS) (*n* = 3)

DED can be induced by environmental factors, age-related factors, and preservatives in eye drops.[21] These factors increase the endogenous and exogenous ROS levels on the ocular surface. Excess ROS can directly or indirectly lead to dysfunction of the corneal and conjunctival epithelia. Therefore, removing excess ROS is a strategy for mitigating dry eye.[22] High-resolution XPS revealed the presence of both Fe^3+^ and Fe^2+^ in the nanozymes. Fe-based nanozymes utilize a unique redox cycle between the Fe^2+^ and Fe^3+^ oxidation states to specifically clear ROS, protecting tissue from damage triggered by excess ROS production. In the presence of excess ROS, borate bonds (named PB in this work) are oxidized to phenol and boric acid, and phenol can be further oxidized.[23] The dual ROS-scavenging properties of our PBnZ nanozyme-based eye drops were attributed to the multienzyme-like activity of the n-Z(Fe) nanozymes and the rapid formation of dynamic phenylboronic acid–diol ester bonds. The ability of PBnZ to eliminate multiple ROS, including H_2_O_2_, O_2_^−^ and OH, in DED was investigated. The mechanisms by which PBnZ scavenges ROS are depicted in Fig. 2a.

### CAT-like enzyme activity of PBnZ

H_2_O_2_ is an important ROS with a strong oxidative capacity that is toxic toward biomolecules.[24] CAT is a common enzyme that eliminates ROS, especially under oxidative stress, by catalyzing the decomposition of H_2_O_2_ and correspondingly producing H_2_O and O_2_.[25] Moreover, Fe ions play a vital role in CAT activity. The reaction equations are as follows:

1 $${\text{H}}_{\text{2}}{\text{O}}_{\text{2}}\text{+2}{\text{Fe}}^{\text{3+}}\text{+2}{\text{H}}^{\text{+}}\rightarrow\text{2}{\text{H}}_{\text{2}}\text{O+4}{\text{Fe}}^{\text{2+}}$$

2 $${\text{H}}_{\text{2}}{\text{O}}_{\text{2}}\text{+4}{\text{Fe}}^{\text{2+}}\rightarrow\text{\text{O}}_{\text{2}}\text{+2}{\text{Fe}}^{\text{3+}}\text{+2}{\text{H}}^{\text{+}}$$

We determined the CAT-like activities of n-Z(Fe), PB and PBnZ by evaluating H_2_O_2_ decomposition and H_2_O_2_ decomposition efficiency. As depicted in Fig. 2b, PBnZ catalyzed the decomposition of H_2_O_2_ the fastest, reaching 80% H_2_O_2_ decomposition within 30 min and showing outstanding catalytic activity. Although n-Z(Fe) and PB were able to decompose H_2_O_2_, their catalytic performances were lower than that of PBnZ and decreased over time. We further explored the catalytic capacity of n-Z(Fe), PB and PBnZ for O_2_ generated from H_2_O_2_. As shown in Fig. 2c, after the addition of n-Z(Fe), PB and PBnZ, the rate of O_2_ production increased with increasing reaction time, but PBnZ generated more O_2_ bubbles than n-Z(Fe) and PB, which proved that PBnZ could effectively catalyze H_2_O_2_ in a CAT-like manner. Additionally, the H_2_O_2_ decomposition reaction of PBnZ followed typical Michaelis-Menten kinetics with a Michaelis-Menten constant (*Km*) and a maximal reaction velocity (*Vmax*). The *Km *and *Vmax* were calculated by fitting Michaelis–Menten and Lineweaver–Burk plots, respectively. The *Km* value indicates the affinity between the catalyst and substrate, and the *Vmax* value of the catalyst refers to the maximum reaction rate in the catalytic process. The relationship between the velocity and the substrate dose, *Km* = 30.687 M and *Vmax* = 41.415 M/s, suggested the strong catalytic oxidation activity of PBnZ (Fig. 2d and Fig. S10). Under the same conditions, we measured and calculated the *Km* and *V*max of n-Z(Fe) (*Km* = 2.642 M, *Vmax* = 6.864 M/s) and PB (*Km* = 19.967 M, *Vmax* = 26.186 M/s) (Fig. S11). These results suggested that PBnZ could efficiently catalyze the conversion of H_2_O_2_ to O_2_ and showed catalase CAT activity. According to the *Km*, the affinity of the three for H_2_O_2_ substrate was in the order of n-Z(Fe), PB and PBnZ, and this result may be related to the porous structure of FeZIF-8, which enabled n-Z(Fe) to fully adsorb H_2_O_2_. The largest *Km* value of PBnZ, indicated the low affinity of PBnZ for H_2_O_2_, which may be due to the smaller particle size of PBnZ. The highest *Vmax* value of PBnZ, indicated that the highest catalytic activity of PBnZ against H_2_O_2_, demonstrated the synergistic action of the nanozyme and the ROS responding borate bond of in PBnZ. At the same time, the *Vmax* value of PB was greater than of n-Z(Fe), indicated that the catalytic activity of PB was higher than that of n-Z (Fe). The enzyme kinetic test were consistent with the CAT-like activity test results described above.

### SOD-like enzyme activity of PBnZ

O_2_^−^, which is persistently generated during the normal metabolism of living organisms, is involved in oxidation/reduction cycles.[26] Fe ions play a vital role in SOD-like activity. The reaction equations are shown in Eqs. (3) and (4):

3 $${\text{O}}_{\text{2}}^{\text{-}}\text{+}{\text{Fe}}^{\text{3+}}\rightarrow\text{\text{H}}_{\text{2}}{\text{O}}_{\text{2}}\text{+}{\text{Fe}}^{\text{2+}}$$

4 $${\text{O}}_{\text{2}}^{\text{-}}\text{+2}{\text{Fe}}^{\text{2+}}\rightarrow\text{\text{O}}_{\text{2}}\text{+}{\text{Fe}}^{\text{3+}}$$

Therefore, we tested the SOD-like activities of n-Z(Fe), PB and PBnZ. Iodonitrotetrazolium chloride (INT) was used as a probe to explore the SOD-like activity of n-Z(Fe), PB and PBnZ. INT is a sensitive superoxide indicator that is reduced by O_2_^−,^ and its absorption peak is typically at 560 nm.[27] As shown in Fig. 2e, in the absence of n-Z(Fe), PB and PBnZ, INT was reduced by O_2_^−,^ with an absorption peak at 560 nm. However, in the presence of these compounds, O_2_^−^ was decomposed by n-Z(Fe), PB and PBnZ, inhibited the reduction of INT by O_2_^−^. The order of SOD-like activity was n-Z(Fe) < PB < PBnZ. In addition, we further explored the SOD-like activity of PBnZ within 30 min, and as shown in Fig. 2f, the SOD-like activity of PBnZ also increased with increasing time in a time-dependent manner. Therefore, PBnZ had stronger SOD-like activity than the other tested materials, which showed that PBnZ nanozymes were ideal catalyst candidates for biomedical applications.

### Ability of PBnZ to scavenge •OH

•OH is the most reactive and most toxic ROS, and enzymatic pathways for eradicating •OH are absent in living organisms.[28] The •OH scavenging reaction is shown in Eq. (5):

5 $${\text{Fe}}^{\text{2+}}\text{+}\text{OH+}{\text{H}}^{\text{+}}\rightarrow\text{\text{Fe}}^{\text{3+}}\text{+}{\text{H}}_{\text{2}}\text{O}\text{ }$$

Thus, the ability of PBnZ to eradicate OH is highly desirable. We used 3,3′,5,5′-tetramethylbenzidine dihydrochloride (TMB) as a probe to determine the •OH elimination efficiency of n-Z(Fe), PB and PBnZ. TMB is a specific and sensitive •OH indicator.[29] OH was produced through the classic Fenton reaction (H_2_O_2_ and Fe^2+^). The chromogenic reaction of TMB and •OH resulted in a color shift from colorless to blue by the oxidation of amino groups to imide groups. The lighter colors indicated that PBnZ could eliminate •OH. As shown in Fig. 2g, the absorption peak at 625 nm was lower than those of n-Z(Fe) and PB, suggesting that, compared with n-Z(Fe) and PB, PBnZ possessed the highest •OH scavenging capacity. Figure 2h showed the high •OH scavenging capacity of PBnZ at a concentration of 200 µg/mL within 30 min, and PBnZ could eliminate more than 62% of the •OH. Taken together, these results revealed the superior ROS scavenging and oxygen production abilities of PBnZ.

In summary, PBnZ was found to have CAT-like and SOD-like activity; CAT can catalyze H_2_O_2_ to generate O_2,_ and scavenge O_2_^•−^ and •OH. n-Z(Fe), PB and PBnZ all exhibited the ability to scavenge ROS. However, PBnZ has the highest multitype enzyme activity because the synergistic mechanism of the n-Z(Fe) nanozymes and borate bond enhanced the multienzyme catalytic activity. The catalytic activity of the Fe coordination complex depends on the electron transfer reaction. The Fe(III) in PBnZ may be oxidized by ROS, leading to the breaking of the coordination bonds and the collapse of the Fe base framework. In addition to Fe(III), which provides electrons, ROS also disrupt the single bond between the boron and the benzene ring, forming an unstable peroxide intermediate, which then hydrolyzes and removes the borate group, and the benzene ring becomes phenol. PBnZ can not only efficiently remove excess ROS to suppress oxidative stress but also degrade them into small ligands and ions to avoid the potential toxicity of long-term retention.

Furthermore, we studied the stability and reproducibility of the abovementioned catalysts. The in vivo stability of the catalysts was simulated by dispersing them into artificial tears, saline, and DMEM (containing 10% FBS) (Fig. 2i). The catalytic activity of PBnZ did not change significantly, indicated the superb stability of the catalysts. These results warranted the further application of PBnZ in the biomedical field.

### In vitro antioxidant and anti-inflammatory activities

The ocular surface is protected by two highly specialized tissues, the corneal and conjunctival epithelia.[30] The biocompatibility of PBnZ is crucial because direct contact with these tissues may result in severe cytotoxicity. Accordingly, we first studied the cytotoxicity of PBnZ to human corneal epithelial cells (HCECs) and conjunctival epithelial cells (CECs) using a cell counting kit-8 (CCK-8) assay. The results showed that the cultured HCECs were tolerant to PBnZ at a concentration of at least 500 µg/mL, and the viability of the cells was maintained at a high level (≥ 80%) (Fig. S12). These results indicated that PBnZ exhibits decreased cytotoxicity and excellent biocompatibility. Moreover, no significant change in the proliferation of HCECs was detected using a CCK-8 assay. These results confirmed the satisfactory biocompatibility of PBnZ with HCECs and CECs under the experimental conditions. Based on the improved biocompatibility of nanozymes, we speculate that the surface-tunable properties of nanozymes provide an opportunity to design biosafety agents. Therefore, in the process of material design, we designed PBA-grafted Fe-based nanozymes and PVA, which are relatively safe for cells. In addition, we speculated that ultrasmall nanozymes can reduce cytotoxicity. When the Fe-based nanozyme was synthesized with n-butylamine, the introduction of n-butylamine reduced its particle size (25 nm). Therefore, we believe that PBnZ eye drops can avoid or reduce potential toxicity.

**Fig. 3:**
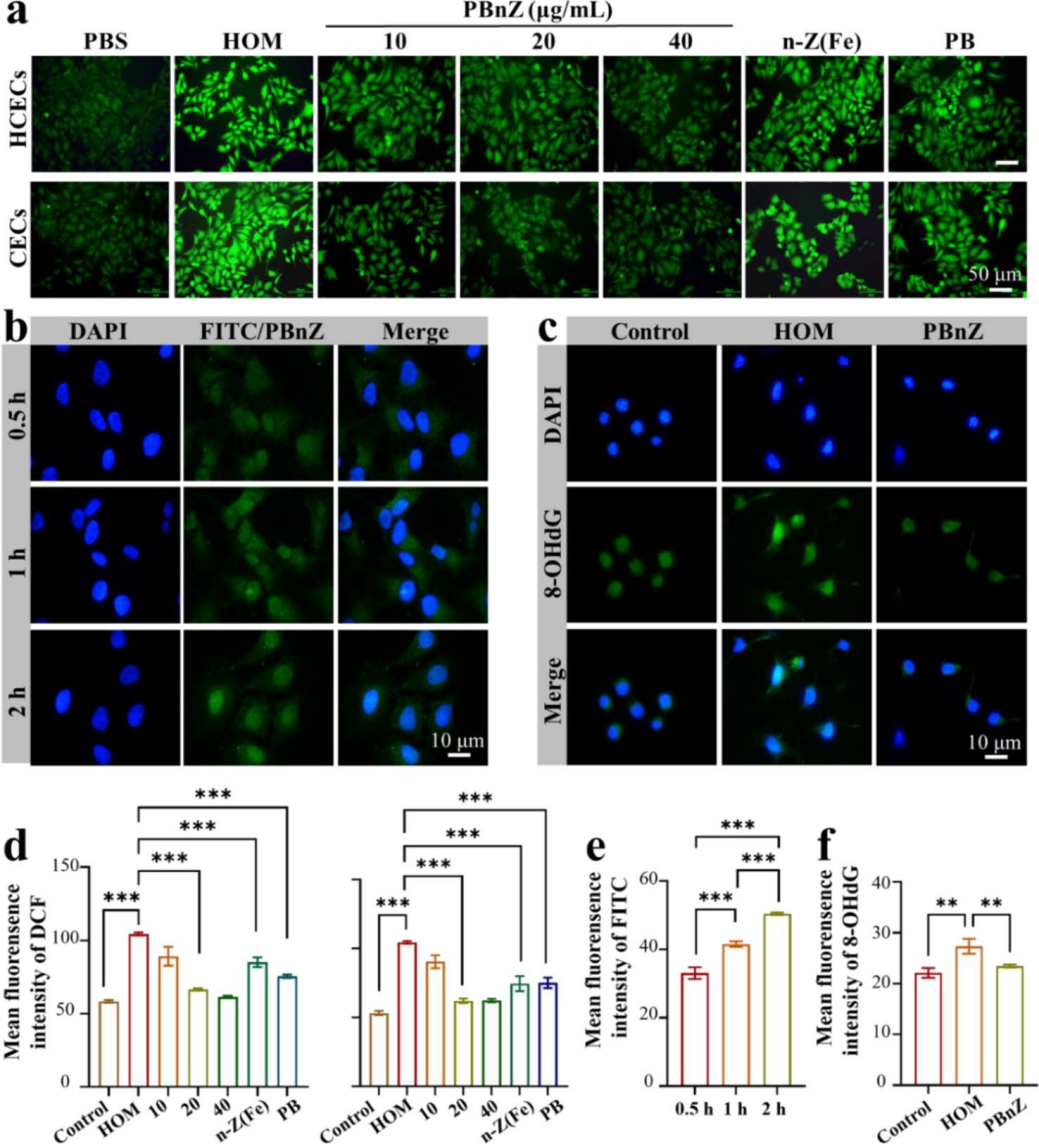
ROS responsiveness and scavenging capability of PBnZ in vitro. (**a**) Intracellular ROS production was measured using DCFH-DA with n-Z(Fe) (40 µg/mL) and PB (40 µg/mL). (**b**) PBnZ was fluorescently labeled with FITC and added to the medium of HCECs for 0.5, 1 or 2 h. (**c**) Immunofluorescence staining of 8-OHdG in hypertonic model HCECs pretreated with PBnZ (20 µg/mL). (**d**) Mean fluorescence intensity of DCF in HCECs and CECs. (**e**) Mean fluorescence intensity of FITC and (**f**) 8-OHdG. (*n* = 3). *The data are presented as the means ± SDs*. *p < 0.05, **p < 0.01, ***p < 0.001 and NS *p* > 0.05

DED can be induced by multiple factors, including environmental factors, age-related factors, and preservatives in eye drops. These factors increase the endogenous and exogenous ROS levels on the ocular surface. Excess ROS can directly or indirectly lead to dysfunction of the corneal and conjunctival epithelium.[31] To determine the ROS scavenging effects of n-Z(Fe), PB and PBnZ in vitro, ROS production was quantified in HCECs and CECs using a DCFH-DA probe under hypertonic conditions (HOM, 500 mOsM), which was selected to induce ROS overproduction and inflammation.[32] Fig. 3a showed the ability of n-Z(Fe), PB and PBnZ to eliminate intracellular ROS in HCECs and CECs induced by HOM. The PBnZ (40 µg/mL) groups displayed fluorescence patterns similar to those of the control groups (PBS), indicated that the former had a remarkable ability to scavenge ROS (Fig. 3d). Although both n-Z(Fe) (40 µg/mL) and PB (40 µg/mL) were able to reduce ROS levels, the effect was more significant in the PBnZ groups.

The cellular internalization of PBnZ in HCECs was further investigated using fluorescein isothiocyanate (FITC)-labeled PBnZ (FITC/PBnZ). As shown in Fig. 3b and e, time-dependent fluorescence signals were detected in HCECs after an incubation with FITC/PBnZ, and strong intracellular fluorescence appeared within only half an hour, indicated rapid and large amounts of cellular uptake of PBnZ. Compared to both the n-Z(Fe) and B@nZ groups with a slow cellular uptake capacity, no or only a very weak fluorescence signal was observed within 0.5 h (Fig. S13). This large uptake of PBnZ may be because PBA and PVA can interact with sialic acid residues at the end of the polysaccharide on the cell membrane surface to form a cyclic boronic acid ester, thereby generating high affinity. Furthermore, the zeta potential of PBnZ can influence its own permeability through the corneal barrier. Positively charged PBnZ enhances permeability, likely due to binding to the negatively charged proteoglycan matrix, which prolongs the corneal residence time of PBnZ. In addition, we confirmed the cytoprotective effect of PBnZ on hypertonic-induced HCEC injury using a CCK-8 assay. Exposure to HOM for 24 h significantly reduced cell viability to an average of 43%, while PBnZ at a concentration of 20 µg/mL improved cell viability to 75% (Fig. S14). PBnZ was shown to have a robust protective effect on apoptosis in HCECs and potential in vivo therapeutic efficacy when exposed to excessive adverse environmental stresses. Therefore, a concentration of 20 µg/mL was selected for subsequent cell-based experiments. Subsequently, the fluorescence of 8-OHdG, an established marker of oxidative PBnZ damage, was markedly elevated in HCECs under HOM but was significantly suppressed by PBnZ (Fig. 3c and f).

**Fig. 4:**
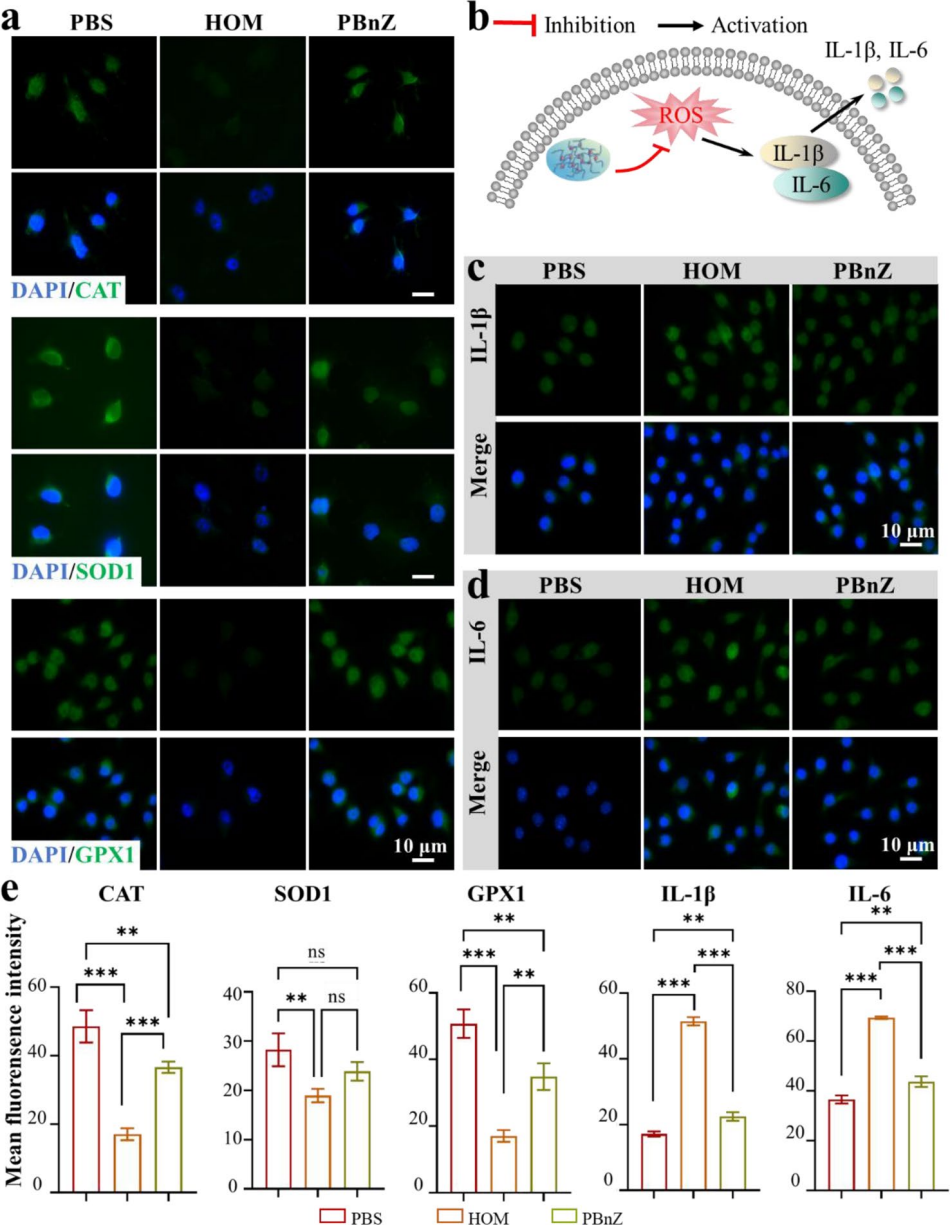
Immunofluorescence staining of PBnZ. HCECs were pretreated with PBnZ (20 µg/mL) before exposure to HOM, as described in the Methods section. (**a**) Immunofluorescence staining of CAT, SOD1 and GPX1. (**b**) Schematic representation of the inhibitory mechanism of intracellular signaling pathways and the targets of PBnZ. (**c**) Immunofluorescence staining of IL-1β and (**d**) IL-6. (**e**) Mean fluorescence intensities of CAT, SOD1, GPX1, IL-1β and IL-6. (*n* = 3). *The data are presented as the means ± SDs*. *p < 0.05, **p < 0.01, ***p < 0.001 and NS *p* > 0.05

Encouraged by the ability of PBnZ to scavenge multiple types of ROS in vitro, the in vivo antioxidant and anti-inflammatory effects of PBnZ were further investigated. First, we observed the antioxidative mechanism of PBnZ under normal physiological conditions. The intracellular antioxidant enzymes included CAT, SOD and glutathione peroxidase (GPX). These compounds can maintain the cellular redox balance, thereby preventing the generation of highly toxic hydroxyl radicals and leading to excess ROS production.[33] We evaluated the expression levels of CAT, SOD1 and GPX1 *via* immunofluorescence staining (Fig. 4a). Consistent with the enzyme activity data, the fluorescence intensities of SOD1, CAT and GPX1 were markedly higher in the PBnZ groups than in the HOM groups (Fig. 4e), indicated that PBnZ effectively counteracted hyperosmolarity-induced reductions in antioxidant enzyme activity.

Thereafter, excessive ROS can activate and trigger a cascade that promotes IL-1β and IL-6 secretion and subsequent ocular surface inflammation (Fig. 4b). We next investigated the potential of PBnZ to inhibit inflammasome activation, given its robust ability to scavenge ROS and perform multiple enzyme-like activities. Immunofluorescence staining revealed that the IL-1β and IL-6 inflammasomes were activated in HCECs exposed to HOM, and these changes were completely abrogated by PBnZ (Fig. 4c and d). Given that the extracellular secretion of IL-1β and IL-6 serves as an indicator of inflammasome activation, we assessed the expression levels of these cytokines in each experimental group using immunofluorescence. PBnZ treatment dramatically reduced the fluorescence intensities of the hypertonicity-induced IL-1β and IL-6 signals.

### PBnZ exhibited extended ocular retention

**Fig. 5:**
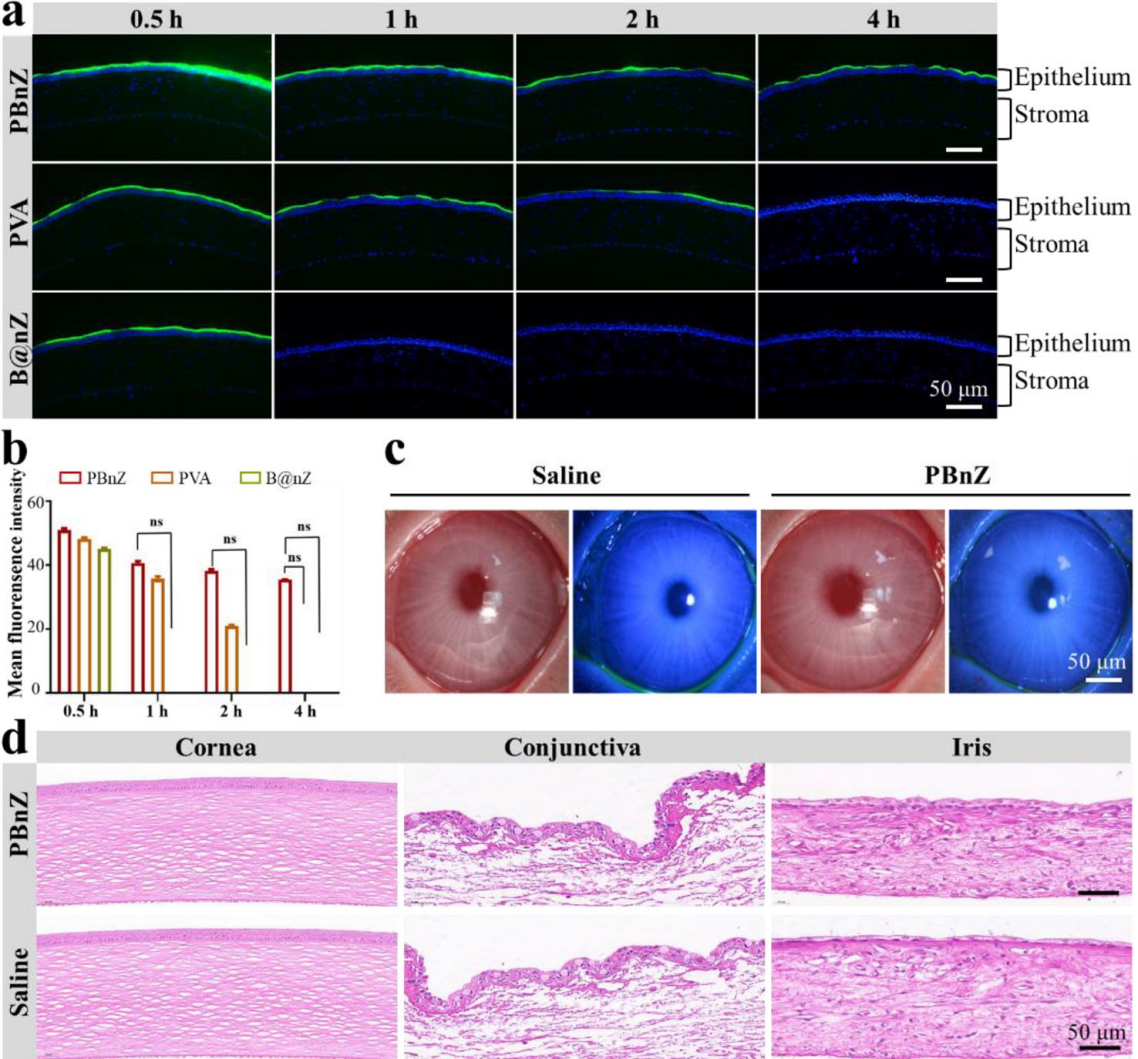
Extended ocular retention and irritation test. (**a**) Representative confocal images of the mouse corneal epithelium at the indicated time points after the administration of PBnZ, PVA or B@nZ. (**b**) Mean fluorescence intensities of PBnZ, PVA and B@nZ. (**c**) Photos of visible and cobalt blue light at 72 h after the administration of a single eye drop. (**d**) Image of H&E staining of ocular tissue (cornea, conjunctiva and iris) 72 h after a single eye drop treatment (left eye: PBnZ; right eye: saline). *The data are presented as the means ± SDs*. *p < 0.05, **p < 0.01, ***p < 0.001 and NS *p* > 0.05

Because PBnZ nanozyme eye drops showed satisfactory biocompatibility and antioxidant and anti-inflammatory effects in vitro, we evaluated acute irritation caused by PBnZ on the ocular surface before performing in vivo animal experiments. No abnormal clinical signs, including corneal defects, opacification, tear turbidity, corneal neovascularization, conjunctival hyperemia, or inflammation, were observed in the eyes of the rabbits after eye drops administration (Fig. S15). Additionally, fluorescein staining revealed no distinct corneal epithelial defects (Fig. 5c). Moreover, the corneal morphology, integrity, and thickness of the epithelium remained normal after topical instillation, as revealed by hematoxylin and eosin (H&E) staining (Fig. 5d). These findings indicate that the PBnZ scaffolds exhibit suitable ocular biocompatibility. The high in vivo biocompatibility and tolerability of PBnZ support its application in alleviating DED.

The long-term retention of PBnZ on the ocular surface could be one of the reasons for its suitable anti-DED performance, which may overcome the low retention of traditional treatments caused by tearing and nasolacrimal drainage.[34] Dynamic covalent complexation between cis-diol and boronic acid moieties has been utilized in numerous biomedical applications. Both PBA-and PVA-tagged micelles can adhere to the ocular surface by forming a dynamic chemical bond with o-diol in the ocular surface mucin.[35] Therefore, we further investigated the preneoplastic effect of PBnZ in vivo. With the assistance of confocal imaging, we observed the presence of FITC-labeled PVA, B@nZ and PBnZ on the corneal epithelium. PVA eye drops were selected as a control group to show that PBnZ eye drops have longer residence times on the ocular surface than traditional eye drops. The main material used in PVA eye drops is polyvinyl alcohol. Its viscosity and osmotic pressure are very close to those of normal tears, and its mucin content, pH and tear composition are basically the same. As shown in Fig. 5a, we strikingly observed a thin and integrated coating consisting of PBnZ on the corneal epithelium, which could be retained for up to 4 h. The relative fluorescence signal of FITC-labeled PBnZ was stronger than that of PVA or B@nZ at the corresponding time points (Fig. 5b), indicated that PBnZ exhibited a longer retention capacity. The specific binding of PBA and PVA to sialic acid residues in mucin is beneficial for prolonging the precorneal residence time. Therefore, the presence of PBA and PVA in PBnZ can play a role in anchoring our eye drops to the ocular surface *via* dynamic covalent complexation with sialic acid moieties. In addition, this difference may have been due to the interaction between PBA and sialic acid residues and the electrostatic interaction between the positively charged amino group in PBnZ and the negatively charged mucin, which enhanced the adhesion of PBnZ to the eyes. This peculiar property could enhance the retention time of PBnZ on the ocular surface and guarantee prolonged and intimate contact with corneal cells, making PBnZ an ideal nanozyme-based eye drop for alleviating DED.

### In vivo therapeutic effects on DED

**Fig. 6:**
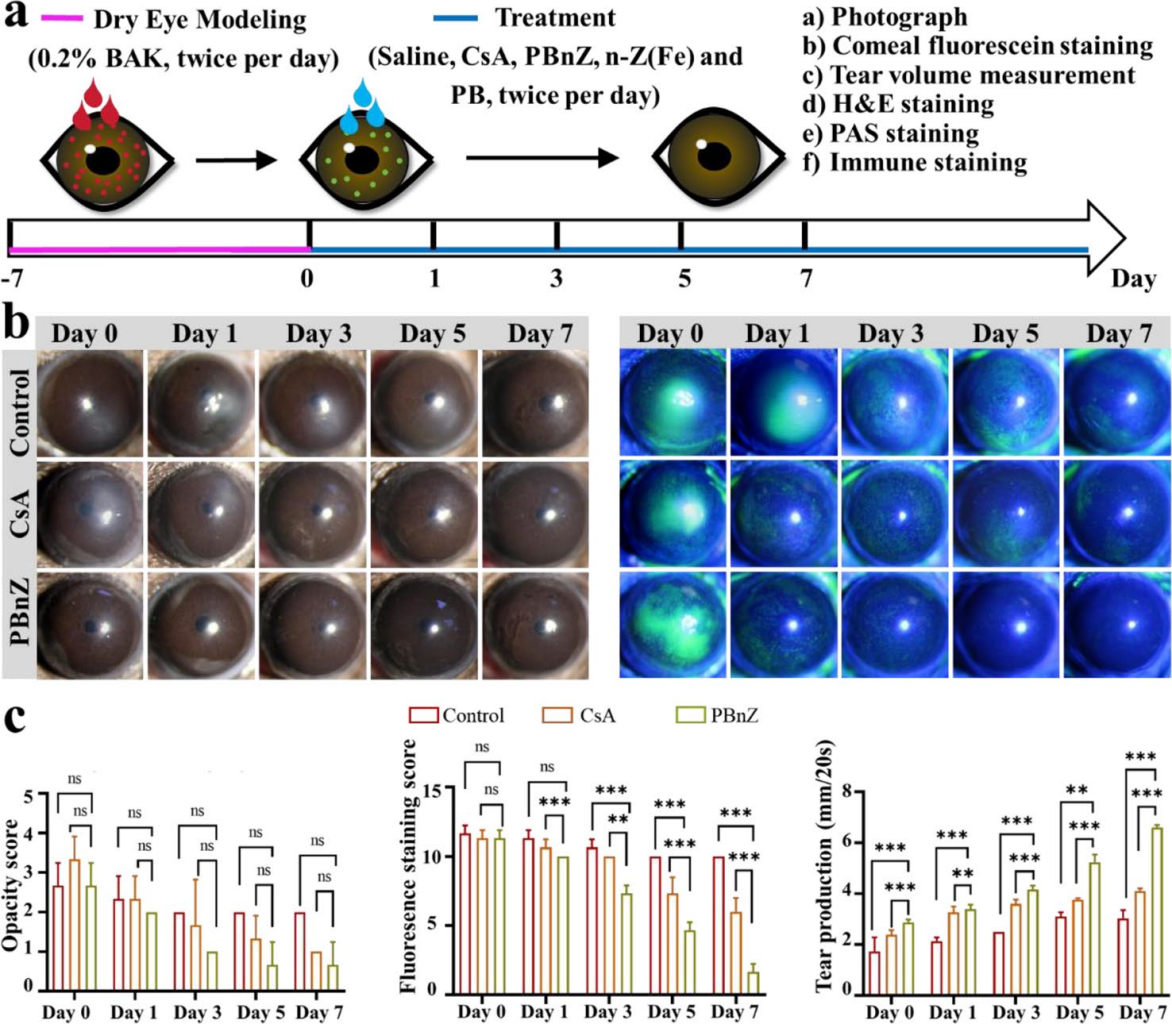
Therapeutic efficacy of PBnZ in mouse model of BAK-induced DED. (**a**) Timeline of the establishment of the dry eye mouse model and treatment administration. (**b**) Optical and corneal fluorescein-stained micrographs of mouse eyes after different treatments. (**c**) Opacity scores, fluorescein staining scores and tear production (mm/20 s) of DED mice treated with saline, CsA and PBnZ. *The data are presented as the means ± SDs*. *p < 0.05, **p < 0.01, ***p < 0.001 and NS *p* > 0.05

The therapeutic efficacy of PBnZ nanozyme-based eye drops for topical ocular treatment was assessed in an experimental benzalkonium chloride-induced DED model. Benzalkonium chloride (BAK) is a widely used preservative that is added to most ocular formulations and can induce DED mainly by causing damage to corneal epithelial cells and accelerating tear film breakdown and evaporation, all of which are accompanied by excess ROS generation.[36] Accordingly, the mice were administered 0.2% BAK eye drops twice a day for 7 days and subsequently received different treatments, including saline (control groups), the commercial eye drops cyclosporine A (CsA, 0.05%), n-Z(Fe) (200 µg/mL), PB (200 µg/mL) or PBnZ (200 µg/mL), twice a day for 7 days (Fig. 6a). These experimental mice had typical clinical symptoms, such as defects on the corneal surface and tear secretion deficiency, indicating successful establishment of the dry eye model.[37] Since the corneal epithelium of normal eyes barely has any defects with negligible fluorescein staining but that of dry eyes results in defects that exhibit positive fluorescein staining, this technique can be used to evaluate the therapeutic effects of DED.[38] After various topical ocular treatments, fluorescein staining of the cornea was monitored photographically via slit-lamp microscopy, and relevant fluorescent vital staining scores were subsequently recorded on a 20-point scale. As shown in Fig. 6b and c, the control groups exhibited strong fluorescence with a continuous staining score > 10.0 for 7 days, indicated severe dry eye symptoms. Treatment with n-Z(Fe) or PB alone (Fig. S16) did not alleviate DED, as characterized by reduced fluorescence. Nevertheless, the residual fluorescein staining of the corneas in the CsA groups, with a score greater than 5.0, suggested that the dry eye-induced corneal epithelial defects were not fully ameliorated on day 7 after monotherapy. Interestingly, after 7 days of PBnZ treatment, the fluorescence in the corneas of the mice decreased gradually, with a final average staining score of 1.5, substantiated a superior therapeutic outcome. Notably, PBnZ had better therapeutic efficacy than CsA. Apart from fluorescein staining, the average tear volume, a common clinical index for evaluating tear stability in DED patients, was used to evaluate DED severity in this study. The average tear volume in DED mice was dramatically reduced to 3.15 ± 0.65 mm, indicated a marked reduction in tear secretion and successful initiation of dryness in the dry eye model mice. No significant changes were detected after treatment with n-Z(Fe) (2.8 ± 0.20) or PB (2.9 ± 0.20), and the average tear volume decreased. However, PBnZ exerted an optimal treatment effect, with a tear volume of 6.15 ± 0.35 mm, which was a better therapeutic effect than that of CsA (4.13 ± 0.10 mm). These results highlight the advantages of PBnZ compared to CsA, as it provides substantial alleviation of DED.

### Histological analyses of the therapeutic effects

**Fig. 7:**
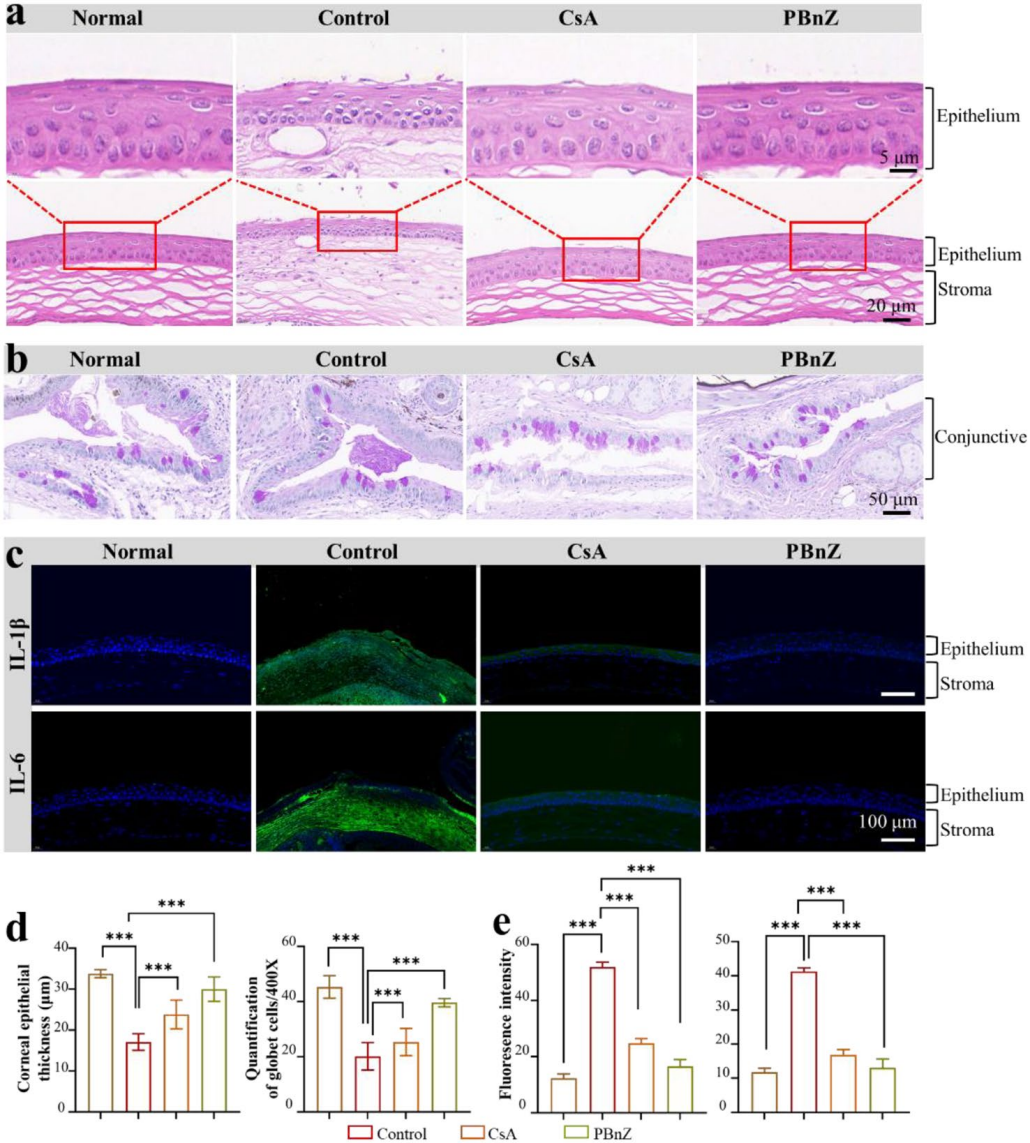
Evaluation of the treatment effect on DED. (**a**) Representative images of H&E staining of the cornea. (**b**) Representative images of conjunctival PAS staining. (**c**) IL-1β and IL-6 expression in the corneal epithelium of the eyes of the mice after the topical administration of saline, CsA or PBnZ for DED was evaluated by performing immunofluorescence staining. (**d**) Quantitative analysis of the corneal epithelial thickness and number of goblet cells in each field. (**e**) Quantitative analysis of the fluorescence intensity of IL-1β and IL-6 expression. *The data are presented as the means ± SDs*. *p < 0.05, **p < 0.01, ***p < 0.001 and NS *p* > 0.05

Inspired by the clinical efficacy of PBnZ, we further conducted histopathological assessments to investigate the structural and morphological changes in ophthalmic organs after various treatments. The overall corneal structure and cell morphology were first evaluated via H&E staining. The normal corneal cells were densely and neatly arranged without inflammatory cell infiltration.[39] However, after DED, the corneal superficial epithelial layers became disrupted and desquamated, the number of epithelial cells decreased, and the morphology became significantly irregular. As shown in Fig. 7a, after treatment with CsA and PBnZ, the corneal cell morphology and structure primarily improved. The corneal epithelial thickness of the normal (33.78 ± 0.98 μm), control (17.10 ± 2.03 μm), CsA (23.82 ± 3.51 μm), and PBnZ (30.02 ± 2.97 μm) groups (Fig. 7d) also confirmed the excellent therapeutic efficacy of PBnZ. Moreover, mucus is a vital component that covers the ocular surface and is secreted mainly by goblet cells. Goblet cells are abundantly distributed within the normal conjunctiva, and the loss of goblet cells is closely associated with DED.[40] As shown in Fig. 7b, periodic acid–Schiff (PAS)-positive goblet cells were abundantly present in the conjunctival fornix of the normal cornea.[41] However, after DED induction, compared with those in the control groups, the number of goblet cells was decreased, and the average area of stained cells in the conjunctiva was significantly lower. In contrast, the pathological changes in the goblet cells improved after treatment with PBnZ. Compared with those in the control groups, the number of goblet cells in the CsA-treated groups was also increased, but the number of goblet cells in the CsA groups was still less than that in the PBnZ groups. The number of goblet cells in the normal group (45.33 ± 4.01) and PBnZ group (39.66 ± 1.53) further confirmed the remarkable efficacy of PBnZ. These results confirmed that PBnZ can critically restore corneal cell morphology and structure and tear secretion in DED model mice.

We investigated inflammatory cytokine levels in ocular surface tissue to further explore the anti-DED mechanism of PBnZ. The effects of different treatments on anti-inflammatory cytokine levels in corneal tissue were assessed using immunohistochemistry.[42] In DED, the elevation of proinflammatory cytokines such as IL-1β and IL-6 can cause damage to the cornea, especially to the epithelial layer.[43] Indeed, after DED modeling, elevated expression of IL-1β and IL-6 was observed on the corneal surface and in the corneal epithelia, respectively. After 7 consecutive days of treatment, the control groups exhibited high IL-1β and IL-6 expression (Fig. 7c), but the CsA and PBnZ groups exhibited lower expression levels. Notably, PBnZ achieved the most effective suppression according to the quantitative analyses of total fluorescence intensity (Fig. 7e). These results confirmed that the decrease in inflammation could be strengthened by ROS scavenging. Compared with commercial eye drops, PBnZ can enhance adhesion to the cornea and thus prolong retention time on the ocular surface, consequently enhancing ROS elimination and greatly contributing to improved efficacy.[44] Taken together, these findings revealed that PBnZ strongly restored ocular surface structures through the inhibition of ROS overproduction and the inflammatory response, suggested that PBnZ was an effective strategy for alleviating DED. Then, gross necropsies and histological analysis of major organs were performed to evaluate the long-term toxicity of PBnZ on day 7 after treatment. As displayed in Fig. S17, no gross or histopathological abnormalities or lesions in the heart, liver, kidney, spleen, or lung were observed.[45] These data revealed that PBnZ has high biocompatibility and excellent ocular tolerance without obvious in vitro cytotoxicity, in vivo systemic toxicity, or corneal toxicity. These findings suggested that PBnZ can be used as a safe nanozyme-based eye drop for future clinical application.

## Conclusion

In conclusion, we successfully developed PBnZ nanozyme-based eye drops with dual ROS-scavenging activity and prolonged corneal retention to remove excess ROS, inhibit oxidative stress, and increase ocular surface retention, which can efficiently alleviate DED. The dynamic borate bonds were modified by amidation reactions to improve the solubility and dispersion of Fe-based nanozymes in solution. The synergistic action of Fe-based nanozymes and borate bonds effectively neutralizes excess ROS (H_2_O_2_, O_2_^•−^ and •OH) in the microenvironment of the ocular surface, thereby reducing oxidative stress and direct oxidative damage. Moreover, PBnZ nanozymes efficiently penetrate ocular tissue and alleviate DED symptoms, with outstanding biocompatibility both in the short-term and long-term for clinical translation. Furthermore, the positively charged PBnZ enhances permeability, likely by binding to the negatively charged proteoglycan matrix, which prolongs the residence time of the formulation on the cornea. In DED model mice, PBnZ treatment significantly alleviated DED by scavenging ROS and increasing ocular surface retention. In addition, the obtained PBnZ exhibited suitable antioxidant and anti-inflammatory activities, strong mucoadhesive properties, and high biocompatibility, leading to desirable therapeutic outcomes for anti-DED without the need for additional drug loading. Therefore, PBnZ is a promising nanotherapeutic strategy for DED alleviation. We strongly believe that the multifunctional PBnZ can alleviate DED and ameliorate ocular surface damage and has great potential as a long-lasting eye drop for alleviating DED in the future and for its future application in the clinic, not only for ocular surface pathologies but also for other inflammation-based diseases.

### Electronic supplementary material

Below is the link to the electronic supplementary material.

Supplementary Material 1

## Data Availability

No datasets were generated or analysed during the current study.
