# Supplementary material for: Dual-ROS-scavenging and dual-lingering nanozyme-based eye drops alleviate dry eye disease
Source: J Nanobiotechnology. 2024 May 8;22:229. doi: 10.1186/s12951-024-02499-0 (PMC11077849; doi:10.1186/s12951-024-02499-0)
Supplement: Supplementary file 1 — Supplementary Material 1 [file 12951_2024_2499_MOESM1_ESM.docx]

***Supplementary Information***

**Dual-ROS-scavenging and dual-lingering nanozyme-based eye drops alleviate dry eye disease**

*Wei Zhang^a,b^, MengyangZhao^a,*^, Dandan Chu^a^, Huiying Chen^a,b^, Bingbing Cui^a,b^, Qingyun Ning^a,b^, Xing Wang^a,b^, Zhanrong Li^a^, ShaokuiCao^b,*^, Jingguo Li^a,b,*^*

^a^Henan Eye Hospital, Henan Provincial People’s Hospital, People’s Hospital of Zhengzhou University, 450003 Zhengzhou, China

^b^School of Material Science and Engineering, Zhengzhou University, 450001 Zhengzhou, China

*Corresponding authors:

E-mail: zhaomyscnu@163.com (Mengyang Zhao)

E-mail: caoshaokui@zzu.edu (Shaokui Cao)

E-mail: cnlijingguo@zzu.edu.cn (Jingguo Li)

**Experimental Section**

**Materials**

2-Methylimidazole (Hmim), zinc nitrate hexahydrate (Zn(NO_3_)_2_·6H_2_O), ferric acetylacetonate (Fe(acac)_3_), n-butylamine, methanol (MeOH), 4-carboxyphenylboronic acid (PBA), N-hydroxy succinimide (NHS), poly(vinyl alcohol) (PVA), fluorescein isothiocyanate (FITC) and 1-ethyl-3-(3-dimethylaminopropyl) carbodiimide hydrochloride (EDC) were purchased from Shanghai Aladdin Biochemical Technology Co., Ltd. (Shanghai, China). 3,3′,5,5′-Tetramethylbenzidine dihydrochloride (TMB), pyrogallic acid, titanous sulfate (Ti(SO_4_)_2_), iodonitrotetrazolium chloride (INT), and FeSO_4_·7H_2_O (≥99.0%) were obtained from Sigma‒Aldrich. All reagents were used as received without further purification. A Cell Counting Kit-8 (CCK-8) was purchased from Dojindo (Japan). 2′,7′-Dichlorofluorescein diacetate (DCFH-DA) and Fluoroshield™ with DAPI were purchased from Sigma–Aldrich (St. Louis, MO, USA). Sodium chloride (NaCl) was purchased from Tianjin Hengxing Chemical Preparation Co., Ltd. The DAPI solution was purchased from Solarbio Science & Technology Co., Ltd. (Beijing, China). SOD1 (bs-10216R), GPX1 (bs-3882R), CAT (bs-6874R), IL-1β (bs-0812R), and 8-OHdG (bs-1278R) antibodies were purchased from Bioss. The FITC-labeled goat anti-rabbit IgG H&L secondary antibody (A0562) was purchased from Absin. Benzalkonium (BAK) chloride was purchased from Hubei Gedian Humanwell Pharmaceutical Excipients Co., Ltd. The TUNEL staining kit was purchased from Roche Co., Ltd., Germany.

**Characterization**

Transmission electron microscopy (TEM) images were obtained using a field emission transmission electron microscope (Tecnai G2 F20 S-TWIN TMP, USA). X-ray diffraction (XRD) measurements were performed using an X-ray diffractometer with a Cu Kα radiation source (45 kV, 40 mA). X-ray photoelectron spectroscopy (XPS) was performed on a K-Alpha spectrometer (AXISSUPRA, Japan). Fourier transform infrared (FTIR) spectra of all polymers were obtained using an IS10 670 FTIR spectrometer in the range between 4000 and 500 cm^-1^ at a resolution of 2 cm^-1^. The UV‒vis analysis was performed using a UV‒visible spectrophotometer (UV-2401PC, Japan Shimadzu) in the range of 190-1100 nm. Dynamic light scattering (DLS) was used to assess the hydrodynamic diameter and zeta potential, which were measured at 25°C using Malvern ZEM 3700 equipment. A surface tension tester (Data Physics OCA20) was used to test the surface tension of the sample, and a rheometer (DHR-1, USA) was used to determine the viscosity at a certain shear rate. The specific surface areas and pore size distributions were surveyed using the Brunauer‒Emmett‒Teller (BET) method based on N_2_ adsorption–desorption isotherms recorded on an ASAP 2460 (Micromeritics, USA) machine at a liquid N temperature (-196°C). The antioxidant properties and fluorescence performance of the samples were investigated with a Cytation5 Microplate Reader (Bio Tek Inc., USA). Fluorescence images were obtained with a Nikon 80i fluorescence microscope.

**Preparation of PBnZ nanozymes eye drops**

Zn(NO_3_)_2_·6H_2_O (1.601 g) and Fe(acac)_3_ (0.7 g) were dissolved in 80 mL of methanol (MeOH). A second solution was prepared by dissolving 2-methylimidazole (3.7 g) and n-butylamine (9.75 mL) in MeOH (80 mL). The resultant n-Z(Fe) was dispersed in pure water (20 mL) by ultrasonic treatment. 4-Hydroxyphenylboronic acid (PBA), N-hydroxy succinimide (NHS) and 1-ethyl-3-(3-dimethylaminopropyl) carbodiimide hydrochloride (EDC) were dissolved in 30 mL of pure water at a molar ratio of 1:1.2:1.2 and activated for 1 h. The previous dispersed n-Z(Fe) solution was added to the activated solution, and the reaction was vigorously stirred at room temperature for 24 h. The resulting precipitate was dried at 60°C for 24 h to obtain PBA-modified n-Z(Fe) (B@nZ). B@nZ was dispersed in PVA (1% w/v) artificial tears by ultrasonic treatment, the pH was adjusted to 8.5, and the final product of the PBnZ nanozyme eye drops was obtained.

**ROS-scavenging ability of PBnZ nanozymes**

The ability of the formulations to scavenge H_2_O_2_ was determined by mixing 25 μL of 100 mM H_2_O_2,_ 25 μL of 200 μg/mL n-Z(Fe), the borate bond (in this work, named PB) or PBnZ in ultrapure water for different durations. The supernatant of the mixture (50 μL) was added to the Ti(SO_4_)_2_ solution (100 μL, separated from 1.33 mL of 24% Ti(SO_4_)_2_ and 8.33 mL of H_2_SO_4_ in 50 mL of ultrapure water). The absorbance of the product TiO_2_ was measured at 420 nm. The decomposition efficiency of H_2_O_2_ was calculated using the following formula:

$$\text{H}_{\text{2}}\text{O}_{\text{2}}\text{ (\%)}\text{=}\frac{\text{A}_{\text{T}}}{\text{A}_{\text{0}}}\text{×100\%} (1)$$

**CAT-like activity of PBnZ**

The CAT enzyme activities of n-Z(Fe), PB and PBnZ were determined at room temperature by measuring the solubility of O_2_ produced at different reaction times using a specific oxygen electrode attached to a dissolved oxygen meter (JPBJ-608, Leici, China). Usually, different concentrations of n-Z(Fe), PB and PBnZ were mixed with H_2_O_2_ in 10.0 mL of purified water. The oxygen solubility (mg/L) was monitored with a dissolved oxygen meter.

**SOD-like activity of PBnZ**

Initially, xanthine (0.6 × 10^–3^ M) and xanthine oxidase (0.05 U/mL) were mixed in 1 mL of phosphate buffer (0.1 M, pH 7.4) to generate O_2_^•−^ for 5 min. Subsequently, different volumes of n-Z(Fe), PB and PBnZ (200 µg/mL) were added, and the mixture was undisturbed for 5 min. Finally, the remaining O_2_^•−^ was detected with an iodonitrotetrazolium chloride (INT) probe. O_2_^•−^ reduced INT to a red product with an absorption peak at 560 nm, which was determined using UV‒vis absorption spectroscopy. The proportion of O_2_^•−^ was calculated as follows:

$$\text{SOD relative activity}\text{ (\% )=}\left[ \frac{\left( \text{A}_{\text{1}}\text{-}\text{A}_{\text{2}} \right)}{\left( \text{A}_{\text{1}}\text{-}\text{A}_{\text{0}} \right)} \right]\text{×100\%} (2)$$

where, A_0_ is the absorbance of INT, and A_1_ and A_2_ represent the absorbance at 560 nm without n-Z(Fe), PB and PBnZ and in the presence of n-Z(Fe), PB and PBnZ, respectively.

**•OH scavenging activity of PBnZ**

In this system, •OH was generated by H_2_O_2_ and FeSO_4_ (0.2×10^−3^ M) after 3 minutes of the Fenton reaction. After treating the solution with different concentrations of n-Z(Fe), PB and PBnZ (100 or 200 μg/mL), the solution was incubated for another 5 minutes to remove the •OH. Finally, the special probe 3,3′,5,5′-tetramethylbenzidine dihydrochloride (TMB, 1×10^−3^ M) was introduced to detect the remaining •OH. UV‒vis absorption spectroscopy was used to quantitate the remaining •OH after TMB was oxidized by •OH. The •OH-scavenging capacity was calculated with Eq. (2). In this equation, A_0_ is the absorbance of TMB, and A_1_ and A_2_ represent the absorbance at 625 nm without n-Z(Fe), PB and PBnZ and in the presence of n-Z(Fe), PB and PBnZ, respectively.

**Cytocompatibility of PBnZ**

The cytotoxicity of PBnZ was detected with a cell counting kit-8 (CCK-8) assay. Human corneal epithelial cells (HCECs) and conjunctival epithelial cells (CECs) were seeded into 96-well plates at a density of 1×10^4^ cells/well and incubated at 37°C with 5% CO_2_ for 24 hours. The cells were divided into ten groups. The culture medium was aspirated, and fresh medium containing different ingredients at various PBnZ concentrations (0, 2, 4, 8, 16, 32, 64, 125, 250 and 500 μg/mL) was added to the wells. After coincubation for 24 or 48 hours, the cells were gently washed with PBS three times. Then, 100 μL of fresh culture medium supplemented with 10 μL of CCK-8 solution was added to each well, and the plates were incubated at 37°C for 4 hours. Afterward, the absorbance of each well at 450 nm was measured using a microplate reader (MDM5, Molecular Devices, San Francisco, CA, USA). The percentage of cell viability was calculated using the following formula:

$\text{Cells viability (\% )=}\left[ \frac{\left( \text{A}_{\text{s}}\text{-}\text{A}_{\text{b}} \right)}{\left( \text{A}_{\text{c}}\text{-}\text{A}_{\text{b}} \right)} \right]\text{×100\% } (3)$

As, Ac and Ab are the experimental, control, and blank groups, respectively.

**Cellular uptake.**

The fluorescent marker FITC was conjugated to PBnZ, B@nZ and n-Z(Fe) to form FITC/PBnZ, FITC/B@nZ, and FITC/n-Z(Fe) to assess the cellular uptake of PBnZ. HCECs were seeded in a 6-well plate with extra glass as the substrate at a density of 1.6×10^3^ cells/glass and cultured under standard conditions for 24 hours. After the removal of the digested medium, the cells were treated with fresh cell culture medium. FITC/PBnZ, FITC/B@nZ, and FITC/n-Z(Fe) were added for 2 hours. Finally, the cells were imaged using a fluorescence microscope.

***In vitro* anti-apoptotic properties**

8-OHdG immunofluorescence staining was performed to evaluate the antiapoptotic effect of PBnZ. Briefly, HCECs were pretreated with PBnZ for 4 hours and then incubated with hypertonic medium (500 mM) for 16 hours. Next, the cells were fixed, permeabilized and blocked with goat serum. Then, the cells were incubated with a primary rabbit anti-8-OHdG antibody at 4°C overnight. After three washes with PBS, the cells were incubated with a FITC-labeled goat anti-rabbit IgG H&L-conjugated secondary antibody for 1 hour and stained with DAPI. The stained slides were monitored under a fluorescence microscope.

**Intracellular ROS elimination effect**

Intracellular ROS generation in human corneal epithelial cells (HCECs) and conjunctival epithelial cells (CECs) was detected using 2′,7′-dichlorofluoresceindiacetate (DCFH-DA). Briefly, HCECs were seeded in six-well plates at the same density and incubated overnight. Then, the cells were pretreated with serum-free DMEM/F12 containing different concentrations of PB, n-Z(Fe) (40 µg/mL) or PBnZ (10, 20 or 40 µg/mL) for 2 hours. Subsequently, the cells were cultured in serum-free DMEM-F12 supplemented with 90 mM NaCl, which was used to create a hypertonic environment (500 mOsm), for an additional 24 hours. The cells were stained for 30 minutes at 37°C with 10 µM DCFH-DA in the dark. Cells cultured under physiological isotonic conditions were used as controls. Each group was observed and photographed under a fluorescence microscope (Olympus, CKX41SF, Japan).

***In vitro* antioxidative properties of PBnZ**

Immunofluorescence staining of CAT, SOD1 and GPX1 was performed to evaluate the antioxidant enzyme activity of PBnZ. HCECs were seeded onto coverslips that had been plated in the wells of a six-well plate at a density of 5 × 10^3^ cells per well and treated with PBnZ (20 μg/mL) as described above. After the cells were fixed with 4% paraformaldehyde (BL539A, Biosharp, China) for 10 minutes, they were washed and permeabilized with 0.5% Triton for 20 minutes and then blocked with goat serum (Elabscience, China) for 30 minutes at room temperature. Then, the HCECs were incubated with primary antibodies against CAT, SOD1 and GPX1 overnight at 4°C. The cells were incubated with a FITC-labeled goat anti-rabbit IgG H&L-conjugated secondary antibody in the dark for 1 hour at room temperature, and the nuclei were counterstained with DAPI. Images were acquired using a fluorescence microscope (Nikon, ECLIPSE80i, Japan).

***In vitro* anti-inflammatory properties**

HCECs were pretreated with or without PBnZ in a hypertonic solution to assess whether the anti-inflammatory effect of PBnZ inhibited inflammasome activation by mitigating oxidative stress. HCECs were washed three times with PBS, fixed with 4% paraformaldehyde for 10 minutes at room temperature, washed with PBS three times, permeabilized with 0.5% Triton for 20 minutes, washed with PBS, and blocked with goat serum for 30 minutes at room temperature. Subsequently, primary antibodies against IL-1β and IL-6 were added and incubated overnight at 4℃. Then, the cells were incubated with FITC-labeled goat anti-rabbit IgG H&L and Cy3-labeled goat anti-mouse IgG (H+L) secondary antibodies for 1 hour at 37℃. Nuclei were counterstained with DAPI. Images were captured using a fluorescence microscope.

**Retention of PBnZ on the ocular surface**

All procedures for animal experiments were performed in accordance with the guidelines of the Chinese Animal Administration and the guidelines of the Association for Research in Vision and Ophthalmology Statement for the Use of Animals in Ophthalmic and Vision Research. Female BALB/c mice weighing 18-20 g and aged 6-8 weeks were used for the *in vivo* study. Then, the mice were randomly divided into three groups (n=6 per group) and received 10 μL of PBnZ or B@nZ and PVA on the ocular surface. FITC-labeled PBnZ ophthalmic fluid was added to the corneal surface of conscious mice. Then, the mice were sacrificed at 0.5, 1, 2, or 4 hours after administration, after which their eyeballs were carefully removed from the orbits and frozen on dry ice with optimum cutting temperature (OCT) (4583, SAKURA) compound. The frozen slices were freshly sectioned and then observed under a fluorescence microscope. The image intensity was calculated using ImageJ software.

***In vivo* evaluation of therapeutic efficiency of PBnZ**

Adult C57BL/6 female mice (6-8 weeks, 20-25 g) were used for animal studies and maintained on a 12-hour light/dark cycle at room temperature with enough food and water. Benzalkonium chloride (0.2%, BAK, Hubei Gedian Humanwell Pharmaceutical Excipients Co., Ltd.) was used to create an experimental DED mouse model. Briefly, each mouse eye was administered 5 μL of 0.2% BAK eye drops twice per day for 7 consecutive days. Then, the mice were randomly divided into five groups (n=6 per group) and treated with 5 μL of a topical eye drop solution of 0.9% sterile saline (control), 200 μg/mL PBnZ, 0.05% Cyclosporine A (CsA, Shenyang Xingqi Pharmaceutical Co., Ltd), 200 μg/mL n-Z(Fe) or 200 μg/mL PB on the ocular surface twice per day for 7 days. Tear volumes were measured using cotton threads (Liaoning Meizilin Pharmaceutical Co., Ltd.) according to the manufacturer’s instructions. The threads were placed on the lower eyelid palpebral conjunctiva at 1/3 of the distance from the lateral canthus. The length of the wetted cotton thread was measured in millimeters at 20 seconds without anesthesia. Corneal opacity was scored using a scale of 0-4 points (0=completely clear; 1=slightly hazy, iris and pupils easily visible; 2=slightly opaque, iris and pupils still detectable; 3=opaque, pupils hardly detectable; and 4=completely opaque with no view of the pupils). Corneal epithelial damage was evaluated using corneal fluorescein staining after sodium fluorescein was instilled into the conjunctival sac, and the cornea was observed under a slit-lamp microscope with a cobalt blue filter. The cornea was divided into five areas (central, nasal, temporal, superior, and inferior), and each area was scored as follows: 0=no staining, 1=slight punctate staining, 2=distinct punctate or slight coalescent staining, 3=distinct coalescent or slight patchy staining, and 4=distinct patchy staining.

**Histopathological Analysis**

After the animals were sacrificed by an anesthesia overdose, the eyes and adnexa were fixed in FAS eyeball fixative (Servicebio, China), embedded in paraffin, cut into sagittal sections (5 μm thick), and then stored at room temperature. Eye sections were stained with hematoxylin and eosin (H&E) for routine histological analysis. Periodic acid–Schiff (PAS) staining was used to observe and calculate conjunctival goblet cell numbers. TUNEL (a marker of apoptosis) immunofluorescence staining was performed using a TUNEL staining kit. The expression of IL-1β and IL-6 in corneal tissues was evaluated by performing immunofluorescence staining. The stained sections were photographed with a digital light microscope (3D HISTECH P250 FLASH, Hungary).

**Statistical analysis**

GraphPad Prism Software Version 6.0 (GraphPad Software Inc., La Jolla, CA) was used to conduct all the statistical analyses. All experiments were repeated at least three times, and the differences between groups were determined using one-way ANOVA. Differences were considered statistically significant at *p<0.05*.


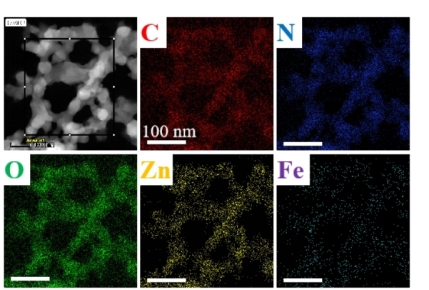


**Figure S1.** Scanning transmission electron microscopy elemental mapping of n-Z(Fe). Scale bar: 100 nm.


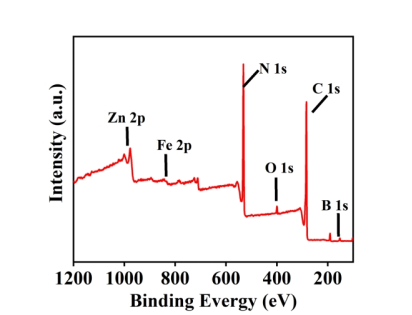


**Figure S2.** XPS spectrum of PBnZ.


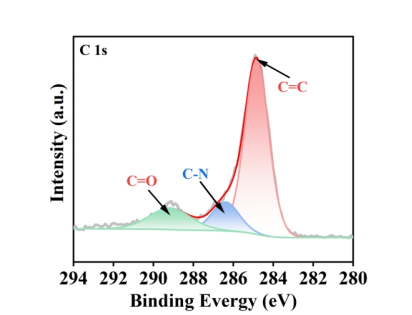


**Figure S3.** XPS C *1s* spectra.


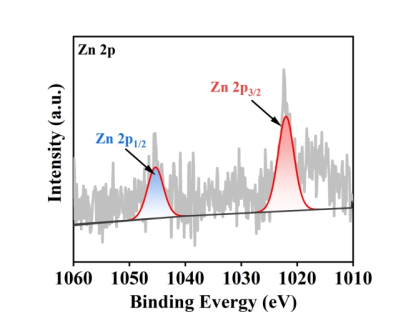


**Figure S4.** XPS Zn *2p* spectra.


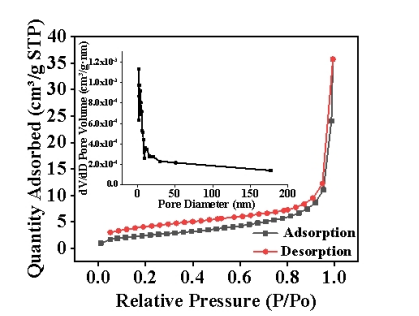


**Figure S5.** An attachment (specific surface) and aperture distribution profile of PBnZ.


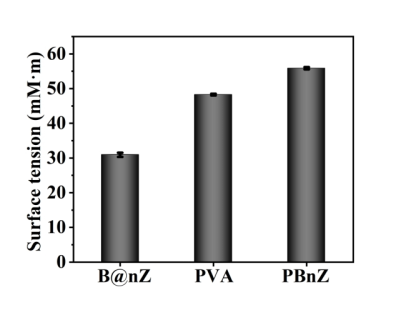


**Figure S6.** Surface tension of B@nZ, PVA and PBnZ.


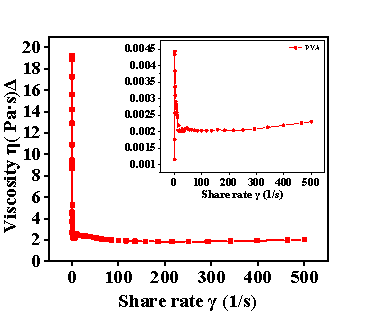


**Figure S7.** Viscosity of PBnZ.


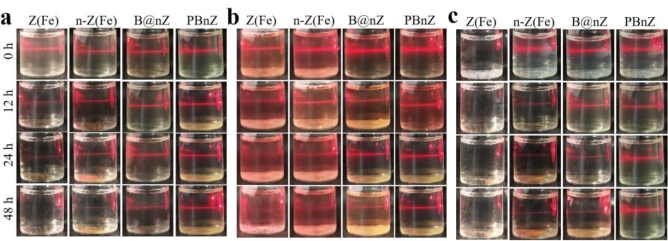
**Figure S8.** Visual view of the scattering stability test of light in artificial tears (a), saline (b) and DMEM (10% FBS) (c).


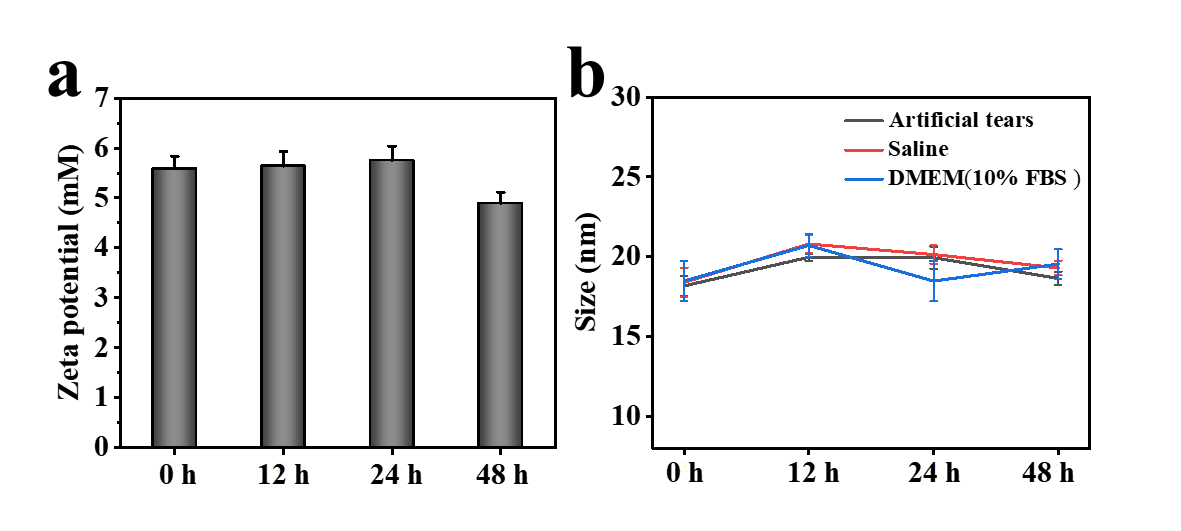


**Figure S9.** (a) Zeta potentials and (b) size of PBnZ during stability testing.


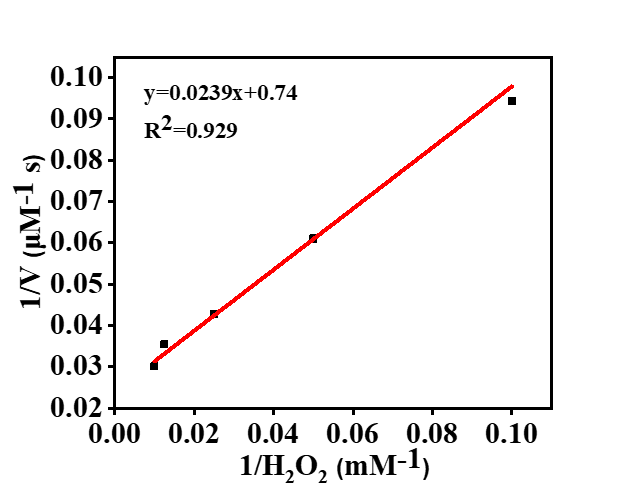


**Figure S10.** Michaelis-Menten kinetic analysis of the CAT-like activity of PBnZ.


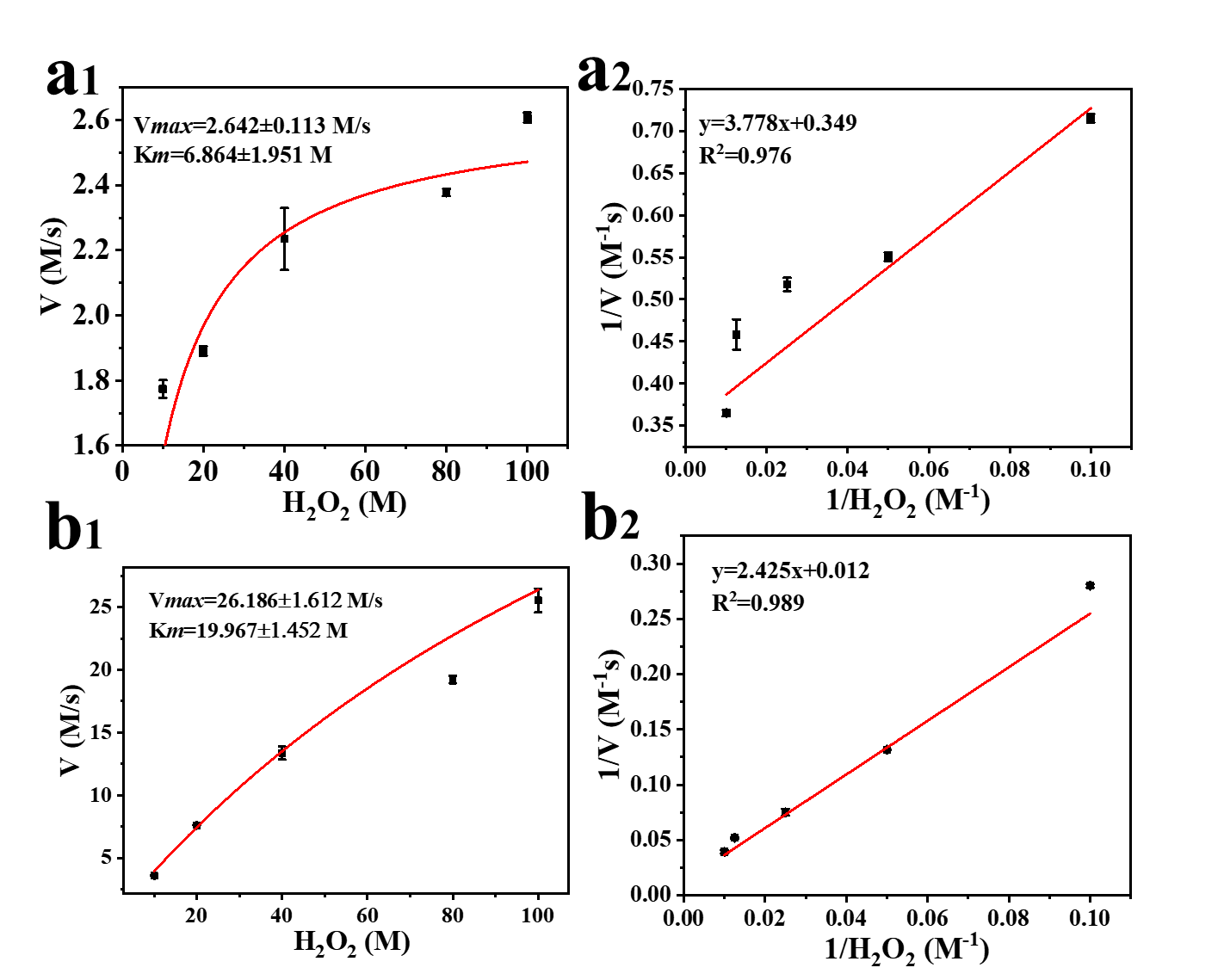


**Figure S11.** Michaelis-Menten fitting curve for the kinetic measurement for n-Z(Fe) (200 μg/mL) (a) and PB (200 μg/mL) (b).


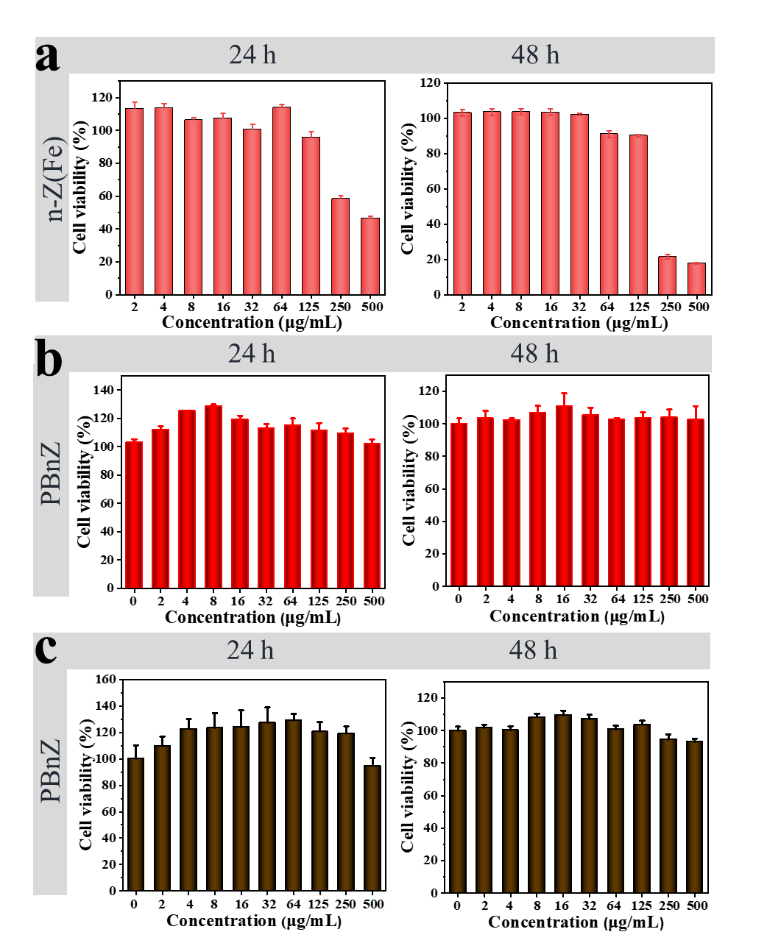


**Figure S12.** (a) Cytotoxicity of n-Z(Fe) toward HCECs. (b) Cytotoxicity of PBnZ in HCECs and CECs.


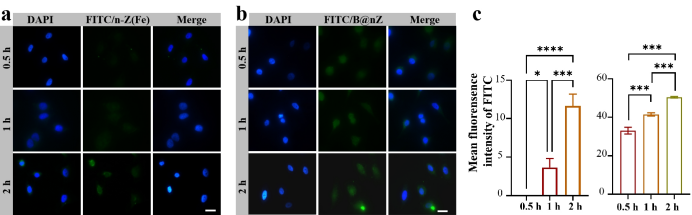


**Figure S13.** (a) n-Z(Fe) and (b) B@nZ were fluorescently labeled with FITC and added to the medium of the HCECs for 0.5, 1 or 2 hours. (c) Mean fluorescence intensity of FITC.


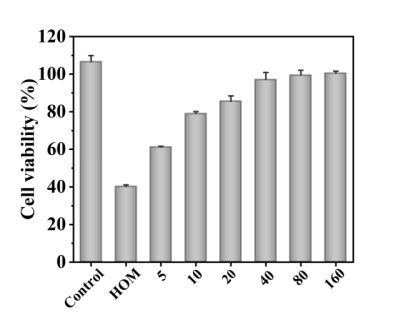


**Figure S14.** Viability of HCECs exposed to a hypertonic model (HOM, 500 mM) and treated with various concentrations of PBnZ.


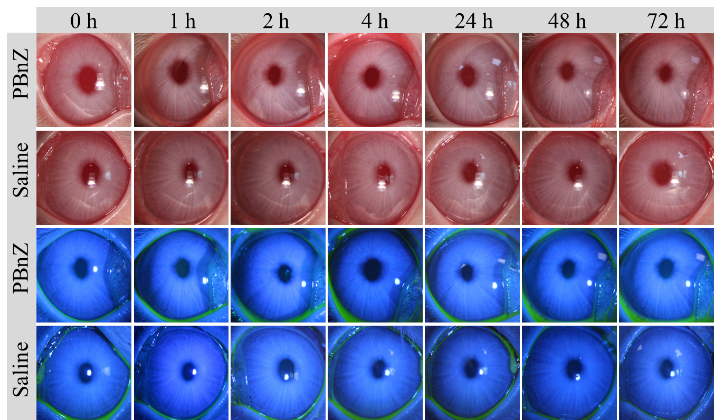


**Figure S15.** Blue light images at different time points of the irritation test (0 h, 1 h, 2 h, 4 h, 24 h, 24 h, 48 h and 72 h).


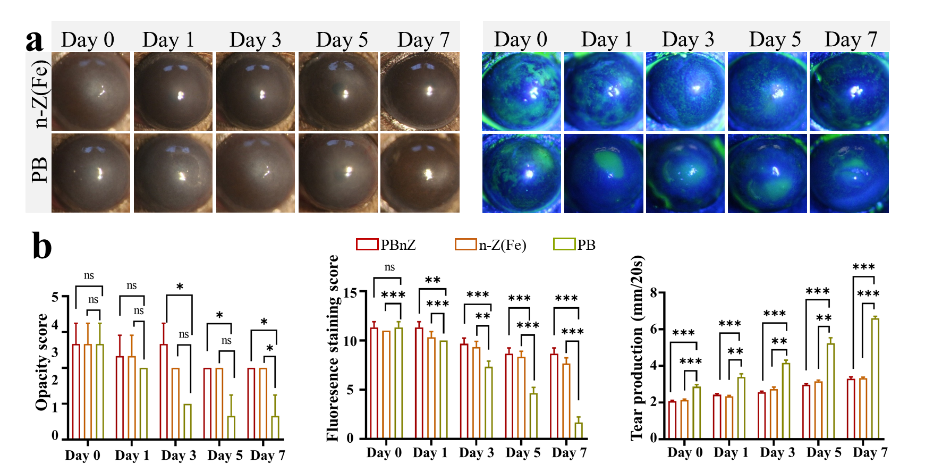


**Figure S16.** (a) Therapeutic efficacy of n-Z(Fe) and PB in a mouse model of BAK-induced DED. (b) Optical and fluorescein-stained corneal micrographs of mouse eyes after different treatments.


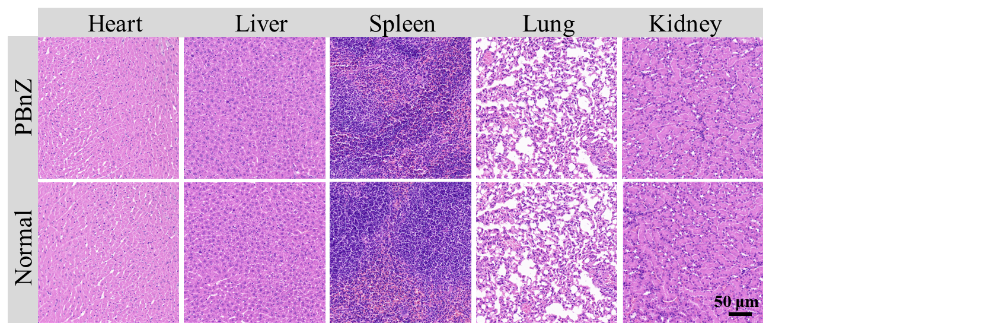


**Figure S17.** *In vivo* biosafety assessment. H&E staining of histological sections of heart, liver, spleen, lung, and kidney tissues.
